# Unlocking a Biological Interface of Chiral Supramolecular Helical Polymers

**DOI:** 10.1021/jacs.5c02902

**Published:** 2025-06-10

**Authors:** Ana Alcalde-Ordóñez, Axel Sarmiento, Jacobo Gómez-González, David Bouzada, Manuel Núñez-Martínez, Manuel Fernández-Míguez, Rafael Rodríguez, Félix Freire, M. Eugenio Vázquez, Miguel Vázquez López

**Affiliations:** † Departamento de Química Inorgánica, Universidade de Santiago de Compostela, 430234 Centro Singular de Investigación en Química Biolóxica e Materiais Moleculares (CiQUS), Rúa Jenaro de la Fuente s/n, 15782 Santiago de Compostela, Spain; ‡ Departamento de Química Orgánica, Universidade de Santiago de Compostela, Centro Singular de Investigación en Química Biolóxica e Materiais Moleculares (CiQUS), Rúa Jenaro de la Fuente s/n, 15782 Santiago de Compostela, Spain; § CINBIO, Departamento de Química Orgánica, Universidade de Vigo, Campus Universitario Lagoas Marcosende, 36310 Vigo, Spain

## Abstract

Here we report a C_3_-symmetric metal-binding tripeptide, **BTMA-1**, that self-assembles in water into either a chiral supramolecular helical polymer or a discrete Co^II^ peptide helicate, depending on metal coordination. The Co^II^ peptide helicate exhibits high affinity and selectivity toward DNA three-way junctions (3WJ), a class of noncanonical DNA structures with emerging biological relevance. Importantly, we demonstrate that the recognition process can be triggered dynamically by adding Co^II^ ions to a dispersion of the supramolecular polymer, which acts as an inert precursor reservoir in physiological media. In this way, our strategy shows that chiral supramolecular helical polymers can form temporarily inactive aggregates that release discrete helicates for biomolecular recognition, such as 3WJ binding, upon metal ion coordination. Overall, this mechanism reveals a previously unexplored capability of this class of materials and offers a new approach for the design of responsive supramolecular systems for nucleic acid recognition and anticancer therapy.

## Graphical abstract

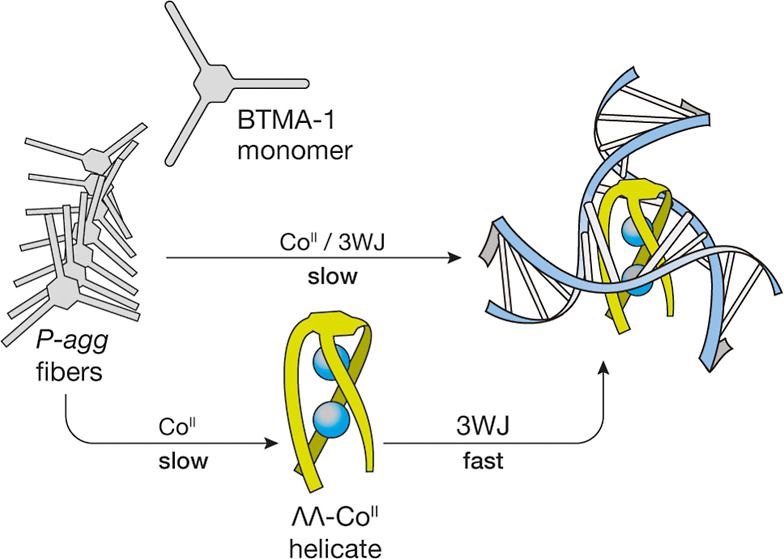

## Introduction

DNA three-way junctions (3WJs) are noncanonical DNA structures that play important biological roles and have emerged as key players in cancer,^1,2^ and promising alternative DNA targets for the development of novel antitumoral agents.^3–7^ 3WJs are symmetric assemblies of three converging double-stranded DNA (dsDNA) branches that intertwine forming a *C*_3_-symmetric hydrophobic cavity lined up by the base pairs at the end of each of the dsDNA strands.^8^ This unique structural feature has been exploited for the design of high-affinity and selective 3WJ binders with matching trigonal symmetry, such as metallocylinders,^9,10^ peptide helicates,^11–15^ azacryptands,^16–18^ triptycenes,^19,20^ cationic calix[3]­carbazoles,^21^ and derivatives with appended peptides for additional nonspecific interactions with the dsDNA arms.^22–24^ In this context, our group has contributed to the development of peptide helicates capable of selectively recognizing and cleaving 3WJs within the nucleus of mammalian cells.^11,15^ Other families of molecules with a *C*_3_-symmetry can also be found in literature, such as the benzene-1,3,5-tricarboxamides (BTAs), which were used by Meijer and others to create supramolecular helical aggregates stabilized by intermolecular hydrogen bonds between amide groups at the periphery and π–π stacking interactions between the central aromatic cores.^25–27^ Notably, C_3_-symmetric BTAs bearing dipeptide arms have been shown to form helical supramolecular polymers in water, with their morphology tuned by the sequence and steric properties of the pendants,^28^ while more recent studies have described stable double-helical supramolecular structures for BTA derivatives in aqueous media, with the helical pitch modulated by subtle changes in monomer structure.^29^ Regarding their practical applications, these supramolecular polymers have been extensively studied during the last decades for their potential uses in drug delivery, biomaterials, and regenerative medicine,^30^ as well as for chiral recognition, enantiomer separation, asymmetric catalysis, and conductive systems, among others.^31^ However, their potential as dynamic reservoirs for bioactive species remains largely unexplored.

We envisioned that a metal-chelating *C*_3_-symmetric tripeptide equipped with a central aromatic core could potentially self-assemble into chiral supramolecular helical polymers through noncovalent interactions or coordinate metal ions to form 3WJ-binding discrete chiral peptide helicates (Figure 1). With this aim in mind, we derivatized a benzene-1,3,5-triyltrimethanamine (BTMA) core with three peptide strands, each containing two metal-binding bipyridine (Bpy) units, to generate the monomer **BTMA-1** (Scheme 1). Thus, while the BTMA aromatic core, the bipyridines, and the amide groups of the peptide strands can support the aggregation of **BTMA-1** monomers into columnar aggregates through π–π and H-bond interactions, the Bpy units of the three peptide strands direct the folding of the **BTMA-1** monomers into discrete helicates upon coordination with Co^II^ ions (Figure 1). The BTMA aromatic core was chosen to ensure sufficient flexibility, allowing the **BTMA-1** monomer to fold into a discrete helicate upon coordination with metal ions. It is important to clarify that, although Bpy ligands can coordinate a variety of metal ions,^11–15^ Co^II^ was selected to minimize undesired oxidation processes in aqueous media and to demonstrate the generation of kinetically inert helicates by in situ oxidation of the metal centers, illustrating the versatility of our approach. Moreover, the role of the chiral l-Arg spacers between the BTMA aromatic core and the bisBpy peptide strands is to define the chirality of the dispersed supramolecular helical polymers as well as to increase the stability of the 3WJ/helicate adducts through salt-bridges with the DNA backbone.^32,33^

**Figure 1:**
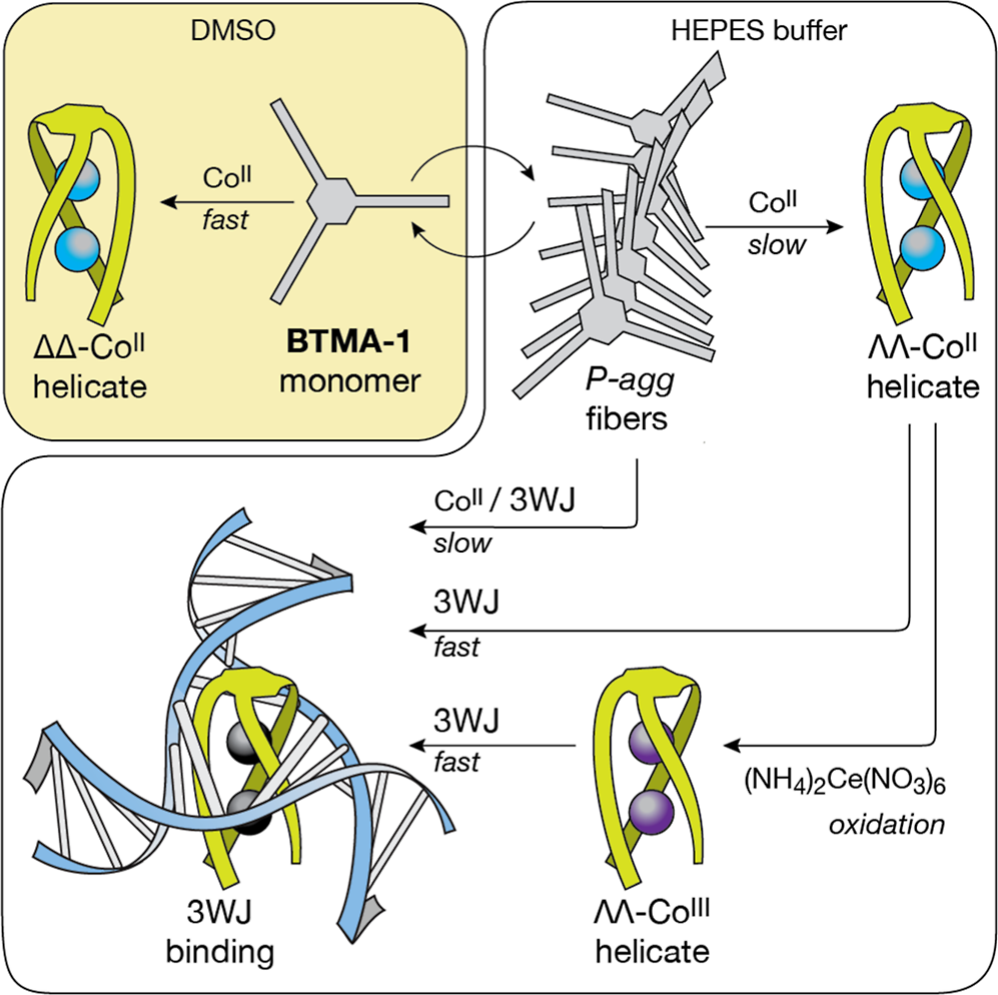
Illustration of the dynamic supramolecular transformations and biomolecular recognition processes described in this work. The **BTMA-1** monomer self-assembles in HEPES buffer into a chiral supramolecular helical polymer (P-agg), which can dissociate in DMSO into soluble monomers. Both the P-agg fibers (in HEPES buffer) and the **BTMA-1** monomers (in DMSO) fold in the presence of Co^II^ ions—although with different kinetics—into discrete peptide helicates with opposite chiralities. The chiral peptide helicate ΛΛ-Co^II^_2_ **BTMA-1** generated in HEPES buffer from P-agg, selectively binds to 3WJs. More importantly, this recognition can also be directly induced by the addition of Co^II^ ions to a dispersion of P-agg fibers in the presence of 3WJs. Finally, ΛΛ-Co^II^_2_ **BTMA-1** can be oxidized in HEPES buffer into its kinetically inert Co^III^ chiral derivative, which also shows selective 3WJ binding properties. The black color of the spheres representing the metal centers of the peptide helicate inserted at the branching point of the 3WJ indicates that these may be two Co^II^ or two Co^III^ ions.

**Scheme 1:**
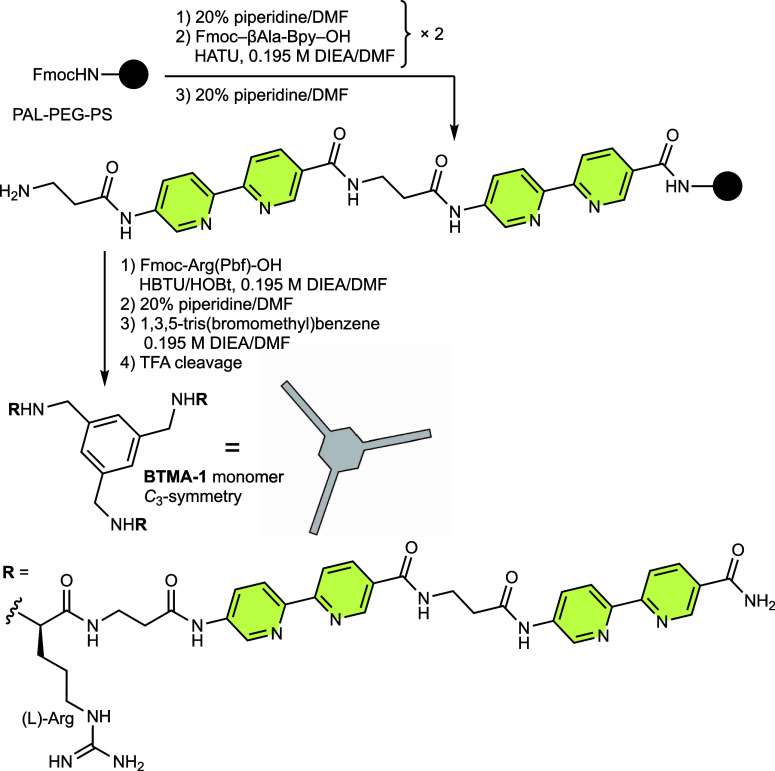
Solid-phase Peptide Synthesis of **BTMA-1** (Peptide Ligand) Monomer

As mentioned earlier, traditional supramolecular helical polymers have been extensively explored for applications in biomaterials and catalysis,^30,31^ yet their potential as dynamic reservoirs for bioactive species remains largely unexplored. Here, we report the first example of a chiral supramolecular helical polymer that acts not only as a ligand source for the formation of discrete helicates but also as a temporarily inactive reservoir that can be selectively activated to release bioactive metallodrugs. This previously unexplored capability of this class of materials enables the dynamic and controlled generation of functional helicates for biomolecular recognition, such as 3WJ binding, through metal ion coordination. Overall, this strategy offers a new approach for the design of responsive supramolecular systems for nucleic acid recognition and anticancer therapy.^9–15^

## Results and Discussion

### Synthesis and Characterization of **BTMA-1**

The peptide ligand **BTMA-1** was synthesized following the procedure in Scheme 1. The H-(l)-Arg-βAla-Bpy-βAla-Bpy-NH_2_ peptide strand was prepared using standard Fmoc- solid-phase peptide synthesis protocols.^34^ Next, three peptide strands simultaneously bound to the BTMA core by on-resin derivatization via a S_N_2 reaction between the N-terminal amino groups and 1,3,5-tris­(bromomethyl)­benzene,^35^ thus generating the **BTMA-1** monomer with *C*_3_-symmetry (Scheme 1). Finally, we performed the combined deprotection and cleavage step with an acidic TFA cocktail and the resulting **BTMA-1** monomer was purified by HPLC and characterized by ESI-MS (Figures S1 and S2).

### Studies on the Chiral Supramolecular Aggregation of **BTMA-1**

The ability of **BTMA-1** to assemble was tested by dissolving it in DMSO, which would keep it in a molecularly dissolved state, or in an aqueous HEPES buffer (10 mM HEPES buffer, NaCl 100 mM, pH 7.5, H_2_O MQ grade), which would induce the aggregation of **BTMA-1** monomers through self-assembly into a columnar polymer. Indeed, while the Electronic Circular Dichroism (ECD) spectrum of a 50 μM solution of **BTMA-1** in DMSO shows no ECD signal, we observe a strong (±) bisignate centered at 310 nm in HEPES buffer at the same **BTMA-1** concentration, which must arise from a chiral arrangement of the Bpy units in the aggregated state (Figure 2a, bottom). Moreover, the UV–vis spectra show a 10 nm hypsochromic shift accompanied by a hypochromic effect of the Bpy π–π* absorption band in HEPES buffer with respect to the one in DMSO (Figure 2a, top). This shift in the Bpy π–π band is accompanied, in HEPES buffer, by the appearance of an additional absorption band centered at λ = 350 nm, which is attributed to the formation of a chiral H-type aggregate.^36,37^ These spectroscopic features, together with the observed formation of extended supramolecular fibers, strongly support the presence of excitonic coupling and π–π stacking interactions between Bpy units along the fiber axis. Such Bpy–Bpy stacking interactions have been previously shown to drive the hierarchical assembly of coiled-coil peptide-based systems bearing Bpy moieties^38^ and discrete metallopeptides,^39^ where they play a key role in promoting the emergence of chiral supramolecular architectures. All these UV–vis and ECD studies are consistent with the formation of a chiral aggregate for **BTMA-1** in HEPES buffer, and the effective solvation of the monomer in DMSO.

**Figure 2:**
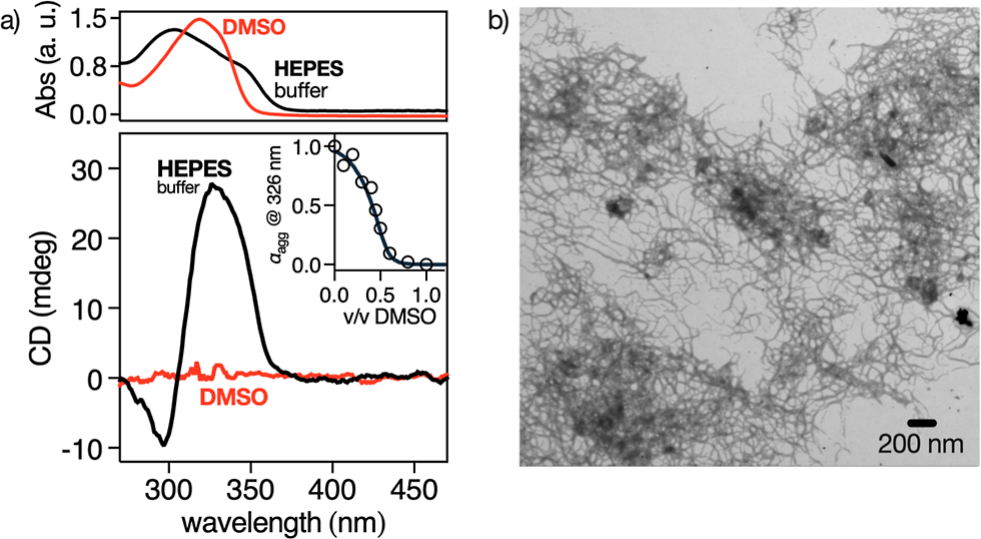
(a) UV–vis (top) and ECD (bottom) spectra of **BTMA-1** in DMSO (orange lines) and HEPES buffer (black lines) at 50 μM of **BTMA-1**. Inset (bottom) shows the ECD solvent denaturation curve with the corresponding fitting (orange solid line) for the **BTMA-1** supramolecular polymer (P-agg) formed at 55 μM of **BTMA-1**. The UV–vis spectra share the same *x*-axis as the ECD spectra shown below to allow direct visual comparison. (b) STEM image showing the fiber-like morphology of P-agg obtained in HEPES buffer ([**BTMA-1**] = 10 μM). Details of the experimental procedure are described in the main text and in the Supporting Information.

We carried out TEM and STEM studies to investigate the aggregate morphology of **BTMA-1** in DMSO and HEPES buffer. These analyses revealed that fiber-like structures are formed exclusively in HEPES buffer ([**BTMA-1**] = 10 μM), consistent with the formation of supramolecular polymers via self-assembly of discoidal **BTMA-1** units into chiral columnar stacks. While the 2D nature of the images obtained by TEM does not allow direct observation of helical features, the elongated morphology observed is in line with previously reported C_3_-symmetric systems that form helical supramolecular aggregates.^40^ Representative STEM and TEM images are shown in Figures 2b and S20, respectively. These data, together with the presence of a positive Cotton band in the ECD spectrum of the aggregate (Figure 2a, bottom), are consistent with the formation of *P*-helical columnar stacks (*P*-agg) dictated by the (l)-chirality of the three arginine residues directly connected to the aromatic core of the **BTMA-1** monomers.

Varying Temperature-ECD (VT-ECD) of *P*-agg in HEPES buffer showed no disassembly of the chiral columnar polymers even at 333 K (Figure S21). The supramolecular association was characterized in more detail using the solvent denaturation (SD) method developed by Meijer and co-workers, which allows us to obtain a complete set of thermodynamic parameters associated with the aggregation process. This method considers the aggregation of **BTMA-1** as a balance between the molecularly dissolved and the aggregated state (*P*-agg), which can be controlled by mixing solutions of the monomer in a “good” (DMSO) and in a “bad” (HEPES buffer) solvent at different ratios. The increase in Gibbs free energy after addition of **BTMA-1**, both in a solvent mixture (Δ*G* ^0^′) and in a pure solvent (Δ*G* ^0^), correlates linearly with the volume fraction of the “good” solvent *f* and the parameter *m*, which indicates the ability of the “good” solvent to interact with the **BTMA-1** monomer, as represented in eq 1

1 $${\Delta G}^{0^{\prime }}={\Delta G}^{0}+m\times f$$

Interestingly, gradual addition of a DMSO solution of **BTMA-1** (molecularly dissolved state) to a dispersion of the same molecule in HEPES buffer (aggregated state, *P*-agg) while keeping its concentration constant in the media, caused a progressive decrease in the intensity of the ECD signal. The experiment was carried out at three different concentrations, [**BTMA-1**] = 55, 50, and 45 μM (Figure 2a, bottom, inset, and Figure S22). The shape of the ECD signal reduction curve and the thermodynamic parameters obtained with the experimental data at 293 K (Δ*G* ^0′^ = −30.5 ± 1.82 kJ/mol; m = 12.4; σ = 2.7 × 10^–2^) are consistent with a cooperative pathway for the supramolecular polymerization process.^41^

Finally, we conducted variable-temperature NMR experiments in D_2_O and DMSO-*d*_6_. The data show progressive broadening and eventual collapse of the NMR signals in D_2_O upon cooling, consistent with aggregation, while sharp, well-resolved signals are maintained across the temperature range in DMSO-*d*_6_, confirming the molecularly dissolved state of **BTMA-1** in this solvent (Figures S32–S34).

### Self-Assembly of the Chiral Co^II^ Peptide Helicates Derived of **BTMA-1**

Having characterized the aggregation of **BTMA-1** monomers in HEPES buffer, we next exploited the quenching of the emission band of the Bpy units contained within the fibers upon metal coordination to study the response of the chiral helical aggregate (*P*-agg) to the presence of Co^II^ ions.^11^ Thus, irradiation at 305 nm of a dispersion of *P*-agg in HEPES buffer ([**BTMA-1**] = 50 μM) resulted in the appearance of an intense emission at 425 nm. This dispersion was titrated with increasing amounts of Co^II^ ions, using Co­(ClO_4_)_2_·6H_2_O as metal ion source, resulting of the progressive quenching of the emission band. The fluorescence data were fitted to a 1:2 (L:M) model in Prism resulting in an apparent dissociation constant of β_D_ ≈ 46.36 ± 1.4 μM (Figure 3a). However, due to the slow coordination kinetics observed during this initial experiment, the titration was subsequently repeated using independently prepared solutions that were incubated overnight to ensure that metal–ligand equilibrium was fully established in each case. Under those conditions we could calculate an apparent 1:2 (L:M) dissociation constant of β_D_ ≈ 0.9 ± 0.6 μM (Figure S4), a value that falls within the range reported for other peptide helicates derived from flexible ligands undergoing entropically demanding folding processes.^11–15^ Notably, the **BTMA-1** Co^II^ helicate exhibits substantially higher stability than miniprotein-based peptide helicates,^12,13^ but moderately lower affinity than oligocationic peptide helicates,^11,14,15^ which benefit from a high degree of preorganization and enhanced charge density. This difference can be rationalized by considering the conformational dynamics of **BTMA-1**, which tends to adopt an extended (open) conformation in aqueous solution and self-assembles into supramolecular fibers. The formation of a discrete dinuclear helicate from such an aggregated precursor involves a thermodynamic entropic penalty, due to the conformational restriction upon folding, but also enthalpic, due to disruption of stabilizing intermolecular interactions within the fibers. In contrast, when the titrations were performed in DMSO, measuring the emission at 388 nm, the fast emission decay of **BTMA-1** could be fitted to a 1:2 (L:M) model in Prism with an apparent dissociation constant of β_D_ ≈ 7.8 ± 0.4 μM (Figure 3c). The binding and kinetic differences observed between both solvents suggest that the stacking between **BTMA-1** monomers in HEPES buffer (aggregated state, *P*-agg), competes with the assembly of the discrete peptide helicates; this competition does not take place in DMSO (molecularly dissolved state).

**Figure 3:**
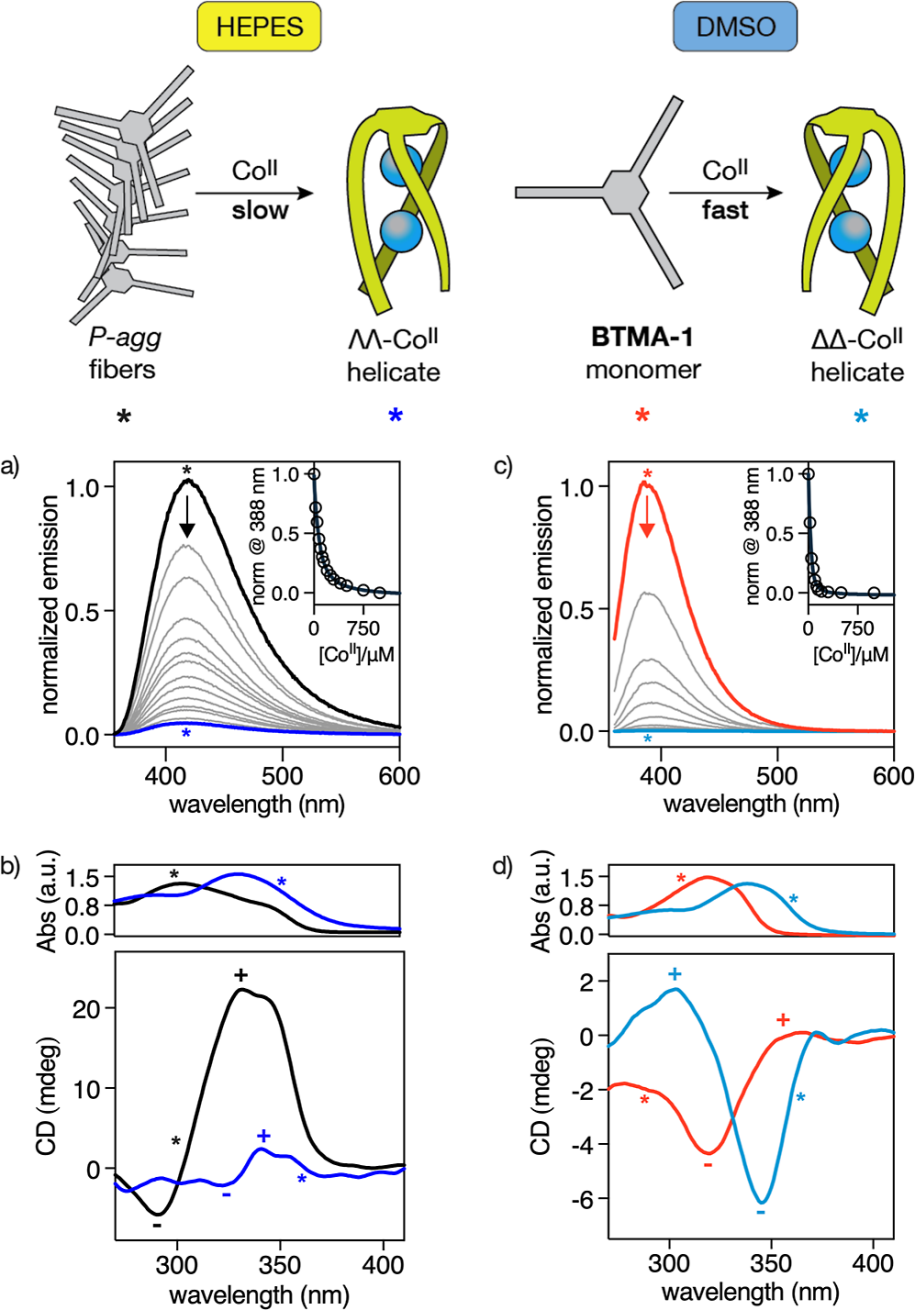
Spectroscopic characterization of the metal-binding equilibria in HEPES buffer and DMSO. (a) Fluorescence titration experiment of P-agg ([**BTMA-1**] = 50 μM) in 10 mM HEPES buffer, 100 mM NaCl, pH 7.0 (black line), with increasing amounts of Co^II^ ions (gray lines of decreasing intensity). Inset shows the titration profile (λ_em_ = 425 nm) of three independent experiments and the best fit according to a 1:2 binding model (L:M) in Prism. (b) UV–vis (top) and ECD spectra (bottom) of a P-agg dispersion ([**BTMA-1**] = 50 μM) in HEPES buffer (black lines) and after 15 min incubation with 15 eq. of Co^II^ ions (blue lines). (c) Fluorescence titration experiment of a 50 μM solution of **BTMA-1** (molecularly dissolved state) in DMSO (orange line), with increasing amounts of Co^II^ ions (gray lines of decreasing intensity). Inset shows the titration profile (λ_em_ = 388 nm) of three independent experiments and the best fit according to a 1:2 binding model (L:M) in Prism. (d) UV–vis (top) and ECD spectra (bottom) of the same 50 μM solution of **BTMA-1** in DMSO (orange lines) after incubation with 15 eq. of Co^II^ ions (light blue lines). In these last cases, the spectral changes were immediately observed. The UV–vis spectra in (b,d) share the same *x*-axis as the ECD spectra shown below to allow direct visual comparison. Details of the experimental procedure are described in the main text and in the Supporting Information. λ_ex_ = 305 nm.

Consistent with these finding, TEM experiments did not show any fibers when a dispersion of *P*-agg in HEPES buffer ([**BTMA-1**] = 10 μM) was mixed with 15 equiv of Co^II^ (Figure S20),^41^ which is consistent with the disassembly of the chiral helical polymer into discrete helicates. Interestingly, addition of Co^II^ to dispersions of *P*-agg in HEPES buffer ([**BTMA-1**] = 50 and 10 μM) slowly led to the progressive disappearance of the UV-band at 354 nm corresponding to the aggregate (*P*-agg), and a slight red shift of the Bpy band, which is consistent with the coordination of Co^II^ ions to the Bpy units of **BTMA-1** (Figure 3b, top, and Figure S6).

In agreement with these observations, the ECD spectra of *P*-agg in HEPES buffer ([**BTMA-1**] = 50 and 10 μM) display intense but slow changes upon incubation with 15 equiv of Co^II^ ions (Figure S5). After 15 min, the spectrum retains a bisignate (±) shape similar to that of the supramolecular polymer, although with lower intensity (from +22.3 mdeg to +2.4 mdeg at the maximum) and a red shift (from 288 to 305 nm), attributed to Co^II^ coordination (Figure 3b, bottom, and Figure S6). Importantly, the sign of the Cotton effect confirms the formation of the discrete ΛΛ-Co^II^_2_ **BTMA-1** peptide helicate in HEPES buffer.

To further confirm the chiral nature of the assembly, we synthesized the enantiomeric ligand of **BTMA-1**, designated as **BTMA-1-D** (Figure S3), which incorporates d-Arg residues in place of the native l-Arg. The ECD spectra of **BTMA-1-D** and its corresponding Co^II^ helicate (ΔΔ-Co^II^_2_ **BTMA-1-D**) were recorded under identical conditions to those used for **BTMA-1** and ΛΛ-Co^II^_2_ **BTMA-1**. As shown in Figure S9, the resulting spectra are perfect mirror images of those obtained with the parent ligand and helicate, thereby confirming the enantiomeric relationship between both systems and the effective transfer of point chirality from the arginine residues to the overall helicate architecture.

In DMSO ([**BTMA-1**] = 50 and 10 μM), the ECD spectrum changes rapidly upon addition of Co^II^, showing an inverted bisignate signal (∓) with a maximum of −1.7 mdeg at 310 nm (Figure 3d, bottom, and Figure S6), consistent with the formation of the ΔΔ-Co^II^_2_ **BTMA-1** helicate. Although less intense than the ECD signal of the aggregate in HEPES, the Co^II^ helicate formed in DMSO exhibits a Cotton effect approximately 6 times stronger than the monomeric **BTMA-1** (−0.1 mdeg), highlighting a notable chiral amplification upon folding. This amplification is likely due to the preferential adoption of a folded conformation, although the exact extent of the folding cannot be determined at this stage. It is known that minor changes in the spatial arrangement of the Bpy ligands, particularly in torsional angles and stacking geometry, can lead to large variations in ECD intensity. As previously reported in related tris­(Bpy) metallopeptides, such distortions can significantly modulate the exciton coupling, even without altering the chiral configuration.^39^ These effects are highly sensitive to solvation, supporting the role of solvent-driven conformational changes in defining helicate chirality.

The formation of a peptide helicate with opposite chirality in HEPES buffer [ΛΛ-Co^II^_2_ **BTMA-1**, ECD (±)] or DMSO [ΔΔ-Co^II^_2_ **BTMA-1**, ECD (∓)] is consistent with a solvent-induced conformational change at the chiral residues (l-Arg) within the **BTMA-1** monomer. This conformational preference may then be transmitted through the peptide scaffold to the bipyridine coordination sites, ultimately determining the helicity of the resulting metal complex. In this context, solvent-induced helix inversion phenomena are well established in chiral foldamers and supramolecular polymers, where external stimuli such as polarity, hydrogen bonding, or redox state can modulate the overall handedness of the system. Recent reviews provide comprehensive insights into how subtle changes in the solvation environment or the folding landscape can trigger inversion of chiral superstructures.^42,43^ Likewise, several examples from metallo-supramolecular chemistry illustrate how solvation and hydrogen-bonding interactions can influence the preferred configuration of metal–ligand assemblies.^44–46^

To assess whether this solvent-driven inversion is reversible, we conducted additional experiments using stock solutions (1 mM) of **BTMA-1** with 15 equiv of Co^II^ prepared in HEPES buffer and DMSO. From these stocks, four samples were prepared at 50 μM **BTMA-1** in (i) pure HEPES, (ii) pure DMSO, and mixed solvents (iii) 95% HEPES/5% DMSO and (iv) 95% DMSO/5% HEPES. The ECD spectra revealed an immediate inversion of the Cotton effect upon switching solvents, confirming that the helicity of the Co^II^ helicate is solvent-dependent and fully reversible (see Figure S7 and Supporting Information, Section 3.3.4).

### Molecular Modeling Studies on the Co^II^ Peptide Helicates in Water and DMSO

Molecular modeling studies of the ΛΛ-Co^II^_2_ **BTMA-1** helicate were carried out using water as the solvent. The computational details are provided in Section 2.10 of the Supporting Information, and the resulting structure is depicted in Figure S23. Notably, when attempting to optimize the structure of the enantiomeric ΔΔ-Co^II^_2_ **BTMA-1** helicate, the system spontaneously reorganized into the ΛΛ configuration, and no local energy minimum corresponding to the ΔΔisomer could be located. This result suggests an intrinsic energetic preference for the ΛΛ configuration under aqueous conditions, in line with our experimental observations.

To further validate this result, we performed molecular modeling of the Co^II^ helicate derived from **BTMA-1-D**, the enantiomeric ligand bearing d-Arg residues. In this case, the minimized structure converged to the expected ΔΔ-Co^II^_2_ **BTMA-1-D** helicate (Figure S30), confirming the mirror-image relationship between the two systems and further supporting the role of the arginine chirality in directing the helicate configuration in water.

Moreover, to investigate the solvent-dependent inversion of helicity, we modeled the Co^II^ helicate derived from **BTMA-1** using DMSO as the implicit solvent. In this case, the global minimum corresponded to the ΔΔisomer (Figure S29), in excellent agreement with the experimental CD data obtained in DMSO. A comparative analysis of the ΛΛ-Co^II^_2_ **BTMA-1** structure in water and the ΔΔ-Co^II^_2_ **BTMA-1** structure in DMSO reveals that, while in water the l-Arg side chains are fully solvated and project outward, in DMSO one of the guanidinium groups establishes intramolecular hydrogen bonding with the amide nitrogen of a neighboring β-alanine residue from an adjacent strand. These specific intramolecular interactions are likely to cancel out the stereochemical control imposed by the L-residues and stabilize the ΔΔconfiguration (Figure S31).

Taken together, the experimental and theoretical results support the notion that solvent polarity and hydrogen-bonding networks govern the folding landscape of the helicate, providing a compelling rationale for the solvent-induced inversion of chirality.

### In Situ Oxidation of ΛΛ/ΔΔ-Co^II^_2_ **BTMA-1** to ΛΛ-Co^III^_2_ **BTMA-1**

Oxidation of ΛΛ-Co^II^_2_ **BTMA-1** to generate the kinetically inert Co^III^ peptide helicate was carried out following previously reported procedures.^11^ A 200 μM aqueous solution of ΛΛ-Co^II^_2_ **BTMA-1** was treated with 16.5 equiv of (NH_4_)_2_Ce­(NO_3_)_6_ (3.3 mM) for 30 min. After purification by HPLC, the formation of the Co^III^ helicate was confirmed by UHPLC-MS (Figure S9) and MALDI-TOF mass spectrometry (Figure S10). ECD measurements of the Co^III^ helicate in HEPES buffer ([**BTMA-1**] = 50 and 10 μM) revealed a bisignate (±) signal similar in shape to that of ΛΛ-Co^II^_2_ **BTMA-1**, confirming the formation of ΛΛ-Co^III^_2_ **BTMA-1** (Figure 4b, bottom). Notably, the metal-to-ligand charge transfer (MLCT) band undergoes a marked bathochromic shift of approximately 50 nm upon oxidation, as observed in both the UV–vis (Figure 4b, top) and ECD spectra. This red shift reflects the enhanced ligand field strength and the altered electronic environment imparted by the Co^III^ centers. This behavior is in line with previous reports on Co^III^ helicates and is further supported by molecular modeling studies, which reveal subtle yet consistent structural differences between the ΛΛ-Co^II^_2_ **BTMA-1** (Figure S23) and ΛΛ-Co^III^_2_ **BTMA-1** (Figure S24) helicates, based on calculations performed in water as the solvent (Figures S25–S28 and Tables S3–S6).^11,47^

**Figure 4:**
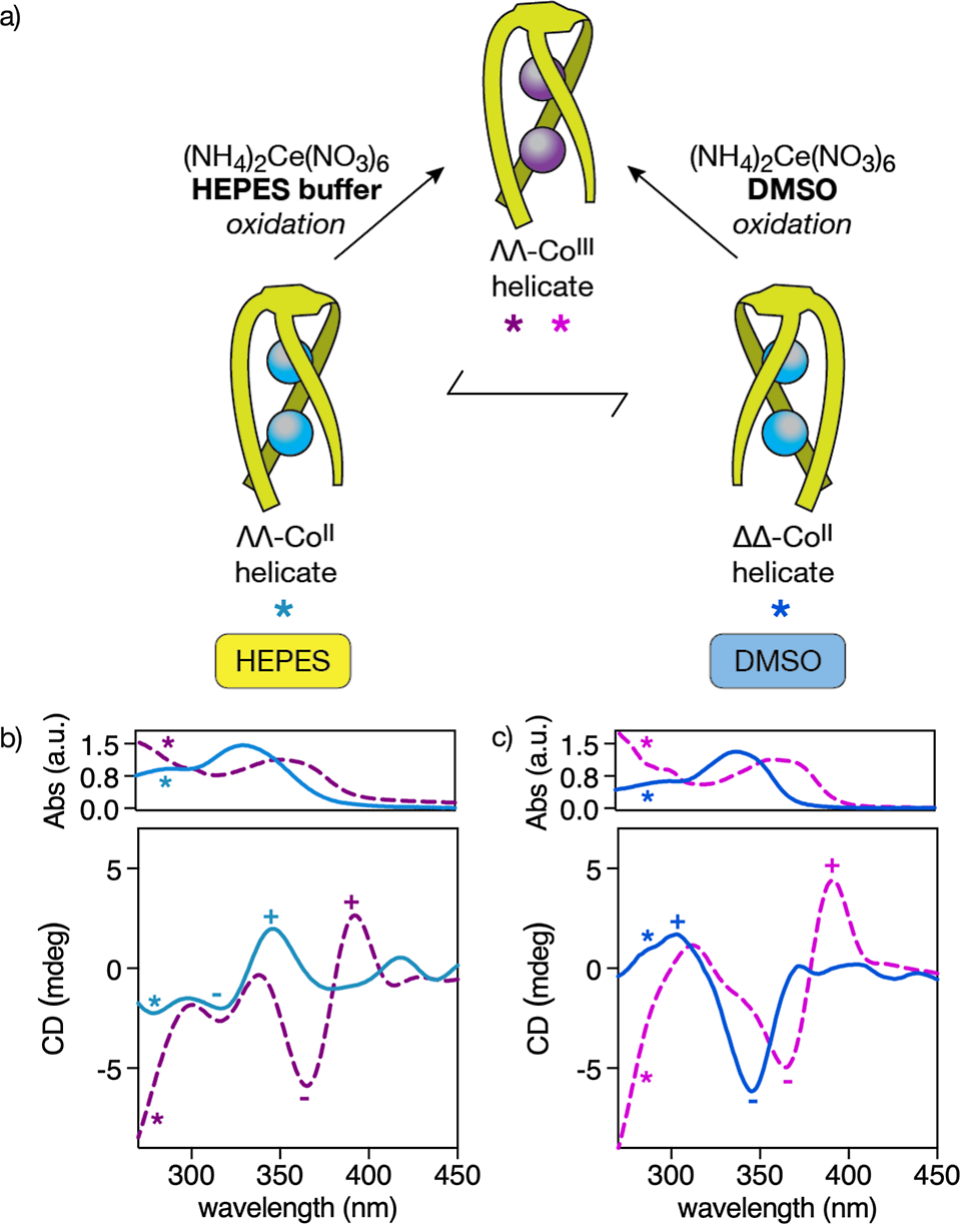
(a) A reversible chiral inversion is observed for the Co^II^ helicate when switching between HEPES and DMSO, whereas an irreversible chiral inversion occurs upon oxidation to the Co^III^ helicate in DMSO, but no in HEPES. (b) UV–vis (top) and ECD (bottom) spectra of 50 μM solutions of ΛΛ/ΔΔ-Co^II^_2_ **BTMA-1** (blue continuous lines in different tones) and ΛΛ-Co^III^_2_ **BTMA-1** (purple dashed lines in different tones) peptide helicates in HEPES buffer (10 mM, 100 mM NaCl, pH 7.0). (c) Same experiments in DMSO. The UV–vis spectra in panels (b,c) share the same *x*-axis as the ECD spectra shown below to facilitate direct visual comparison. Details of the experimental procedures are provided in the main text and in the Supporting Information.

Similar oxidation experiments were carried out with the ΔΔ-Co^II^_2_ **BTMA-1** helicate obtained in DMSO (Figures S12 and 4c). Interestingly, we observed in this case an inversion of the band from ∓ to ± during the oxidation to Co^III^ with (NH_4_)_2_Ce­(NO_3_)_6_, indicating a chiral inversion, from ΔΔ-Co^II^_2_ **BTMA-1** to ΛΛ-Co^III^_2_ **BTMA-1**. The inversion of helicity upon oxidation in DMSO, but not in HEPES, suggests a critical role of solvation and local environmental polarity in determining the final chiral configuration of the helicate. While the coordination geometry of the metal centers should remain preserved, we hypothesize that the combination of the oxidizing agent [(NH_4_)_2_Ce­(NO_3_)_6_], solvent-specific solvation effects, and the increased rigidity of the Co^III^ coordination sphere induces subtle conformational rearrangements in the **BTMA-1** ligand. We suggest that, in the same way as in the Co^II^ analogues described above, changes in the conformation of the l-Arg side chains could be transmitted to the achiral Bpy units and ultimately influence the overall helicity of the Co^III^ peptide helicate. These results highlight the potential of metal ion oxidation to induce chiral inversion, offering a new strategy for the design of structurally defined, redox-responsive chiral architectures.

Finally, it should be noted that the kinetically inert Co^III^ peptide helicates were obtained by complete oxidation of their Co^II^ analogues in both solvents, isolated by preparative HPLC, and fully characterized by UHPLC-MS and MALDI-TOF. This ensures that all studies were performed with pure and well-defined dinuclear Co^III^ metallopeptides (Supporting Information, Section 3.4, and Figures S10–S12).

### Recognition of 3WJ by ΛΛ-Co^II^_2_ **BTMA-1** and ΛΛ-Co^III^_2_ **BTMA-1**

Once we obtained the Co^II^ and Co^III^ chiral peptide helicates derived from **BTMA-1**, we studied their 3WJ binding properties by monitoring the fluorescence quenching of a 2 μM solution of a fluorescein-labeled 3WJ (λ_em_ = 515 nm; λ_exc_ = 490 nm; 3WJ-FAM) in HEPES buffer (10 mM, 100 mM NaCl, pH 7.0) upon addition of increasing amounts of the preformed ΛΛ-Co^II^_2_ **BTMA-1** (Figure 5a) or ΛΛ-Co^III^_2_ **BTMA-1** (Figures 5b and S15). The resulting titration profile at 515 nm for ΛΛ-Co^II^_2_ **BTMA-1** could be fitted to a 1:1 plus nonspecific binding model (3WJ:helicate) in *DynaFit*,^11–15,48,49^ resulting in an apparent *K*_D_ of 360 ± 17 nM (Figure 5a, inset), which agrees with the affinity showed by other peptide helicates.^11^ Regarding ΛΛ-Co^III^_2_ **BTMA-1**, the titration profile at 515 nm was fitted to a mixed 1:1 + 1:2 + other nonspecific interactions model (3WJ:helicate) in *DynaFit*.^11^ This model is characterized by three different dissociation constants, *K*_D1_ = 111 ± 55 nM, *K*_D2_ = 4.5 ± 2.1 μM and *K*_D3_ = 10.8 ± 8.8 mM likely corresponding to the specific insertion of the Co^III^ peptide helicate into the 3WJ branching point, nonspecific interactions with the B-DNA arms of the 3WJ and aggregation processes due to other nonspecific interactions, respectively (Figure 5b). Notably, although *K*_D1_ for the Co^III^ helicate indicates a higher specific affinity for the 3WJ core, the Co^II^ helicate engages in a more selective interaction, dominated by a single 1:1 complex with the 3WJ structure and showing fewer contributions from nonspecific binding or aggregation processes. We could not determine the *K*_D_ of the binding of the Co^II^ and Co^III^ peptide helicates to canonical double-stranded DNA because the working mixtures precipitated before reaching the end of the titration, pointing to nonspecific electrostatic interactions between the chiral peptide helicates and B-DNA (Figures S13 and S14).^11^

**Figure 5:**
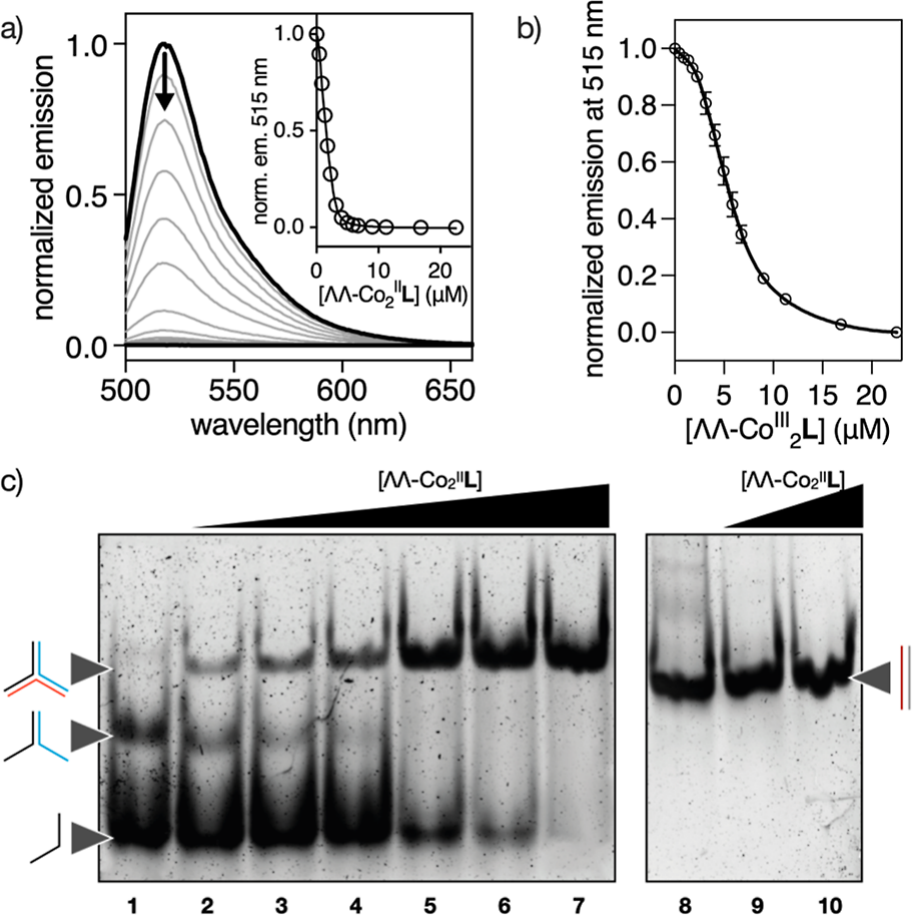
(a) Normalized emission spectra of a 2 μM solution of 3WJ-FAM in absence (thick black line) and in the presence of increasing amounts of ΛΛ-Co^II^_2_ **BTMA-1** (black lines of decreasing intensity); inset shows the titration profile at 515 nm of the same experiment with the best fit to a 1:1 + nonspecific interactions (3WJ:helicate) binding model in DynaFit (thick line; **L** = **BTMA-1**). (b) Profile at 515 nm of the titration of a 2 μM solution of 3WJ-FAM with increasing amounts of ΛΛ-Co^III^_2_ **BTMA-1** with the best fit to a 1:1 + 1:2 + nonspecific interactions (3WJ:helicate) binding model in DynaFit (thick line; ; **L** = **BTMA-1**). Conditions: HEPES buffer (10 mM, 100 mM NaCl, pH 7.0); λ_exc_ = 490 nm; 3WJ-FAM oligonucleotide sequences: Y1:5′–FAM-TTTT CAC CGC TCT GGT CCT C–3′; Y2:5′–CAG GCT GTG AGC GGT G–3′; Y3:5′–GAG GAC CAA CAG CCT G–3′. (c) Left, gel displacement assays of 200 nM of 3WJ in the absence (lane 1) and in the presence of increasing concentrations of ΛΛ-Co^II^_2_ **BTMA-1**: 25, 50, 100, 250, 500, and 750 nM (lanes 2 to 7). Please note that in the absence of the peptide helicate (lane 1), the 3WJ structure does not assemble, and only bands corresponding to single- and partially double-stranded oligonucleotides are observed. Right, gel displacement assays of 200 nM of B-DNA in the absence (lane 8) and in the presence of increasing concentrations of ΛΛ-Co^II^_2_ **BTMA-1**: 500 and 1000 nM (lanes 9 and 10). 3WJ oligonucleotide sequences: Y1:5′–CAC CGC TCT GGT CCT C–3′; Y2:5′–CAG GCT GTG AGC GGT G–3′; Y3:5′–GAG GAC CAA CAG CCT G–3′. B-DNA oligonucleotide sequences: Z1:5′–AAC ACA TGC AGG ACG GCG CTT–3′; Z2:5′-AAG CGC CGT CCT GCA TGT GTT-3′. Details of the experimental procedure are described in the main text and in the Supporting Information.

To further characterize the DNA binding properties of ΛΛ-Co^II^_2_ **BTMA-1** and ΛΛ-Co^III^_2_ **BTMA-1**, we performed electrophoretic mobility shift assays (EMSA) in polyacrylamide gel (PAGE) under nondenaturing conditions.^50,51^ Upon incubation of the target 3WJ with increasing concentrations of either the preformed Co^II^ or Co^III^ peptide helicates, a new, slower-migrating band appeared in a concentration-dependent manner, accompanied by a concomitant decrease in the intensity of the original bands corresponding to the free oligonucleotides. Notably, in the absence of the peptide helicate, no 3WJ assembly is observed, and only bands associated with single-stranded and partially (double-stranded) hybridized oligonucleotides are detected. These changes are consistent with the formation of the expected 3WJ/ΛΛ-Co^II^_2_ **BTMA-1** and 3WJ/ΛΛ-Co^III^_2_ **BTMA-1** adducts (Figures 5c and S17, respectively). Interestingly, no smearing is observed, even at high helicate concentrations, indicating the formation of a single, well-defined helicate:3WJ complex. In contrast, incubation of ΛΛ-Co^II^_2_ **BTMA-1** or ΛΛ-Co^III^_2_ **BTMA-1** with a model B-DNA did not generate any new bands (Figures 5c and S17, respectively), thus confirming the poor affinity of these chiral discrete complexes for canonical DNA. To further validate this selectivity, we performed additional competitive EMSA experiments using both helicates in the presence of 3WJ and B-DNA (Figures S18 and S19). These results confirmed that both helicates preferentially bind to the 3WJ structure, with no observable interaction with B-DNA under the same conditions, supporting the high binding selectivity of our systems toward this noncanonical DNA motif. Altogether, the EMSA data demonstrate that both ΛΛ-Co^II^_2_ **BTMA-1** and ΛΛ-Co^III^_2_ **BTMA-1** recognize with high affinity and selectivity 3WJs over canonical B-DNA in vitro.

### Recognition of 3WJ Directly from the Chiral Supramolecular Helical Polymer (P-agg)

Finally, we explored the potential of the chiral aggregate (*P*-agg) to act as a reservoir of **BTMA-1** ligands and enable the in situ assembly of the ΛΛ-Co^II^_2_ **BTMA-1** helicate in the presence of Co^II^ ions and the recognition of 3WJ in one pot. As a reference of the 3WJ binding kinetics, we first prepared a stock dispersion of *P*-agg ([**BTMA-1**] = 100 μM) in 10 mM HEPES buffer 100 mM NaCl, pH = 7.0, and allowed this to incubate overnight in the presence of 15 equiv of Co^II^ ions to induce the complete disassembly and formation of the ΛΛ-Co^II^_2_ **BTMA-1** helicate. Then, a 2 μM solution of a fluorescein-labeled 3WJ (3WJ-FAM) was incubated with 3.75 equiv of the ΛΛ-Co^II^_2_ **BTMA-1** helicate from this solution, and monitored over time the emission intensity of fluorescein at 325 nm; the resulting intensity profile shows a rapid decrease consistent with the formation of the ΛΛ-Co^II^_2_ **BTMA-1**/3WJ-FAM complex with a half-life of less than a minute (Figure 6b, process 1). In contrast, when the same experiment was repeated adding, over a mixture of 2 μM 3WJ-FAM plus 56.25 equiv of Co^II^ in HEPES buffer, 3.75 equiv of the same *P*-agg dispersion used before ([**BTMA-1**] = 100 μM), we observed that the emission intensity of 3WJ-FAM decreased very slowly with a half-life of about 1 h, which is consistent with a much slower 3WJ binding than that observed in the previous experiment with the preformed helicate (Figure 6b, process 2). These experiments demonstrate the ability of **BTMA-1** monomers to remain biologically inactive as a chiral helical polymeric aggregate (*P*-agg) in physiological media and to transform into bioactive discrete chiral molecules (ΛΛ-Co^II^_2_ **BTMA-1**) upon exposure to an external stimulus, specifically metal ions.

**Figure 6:**
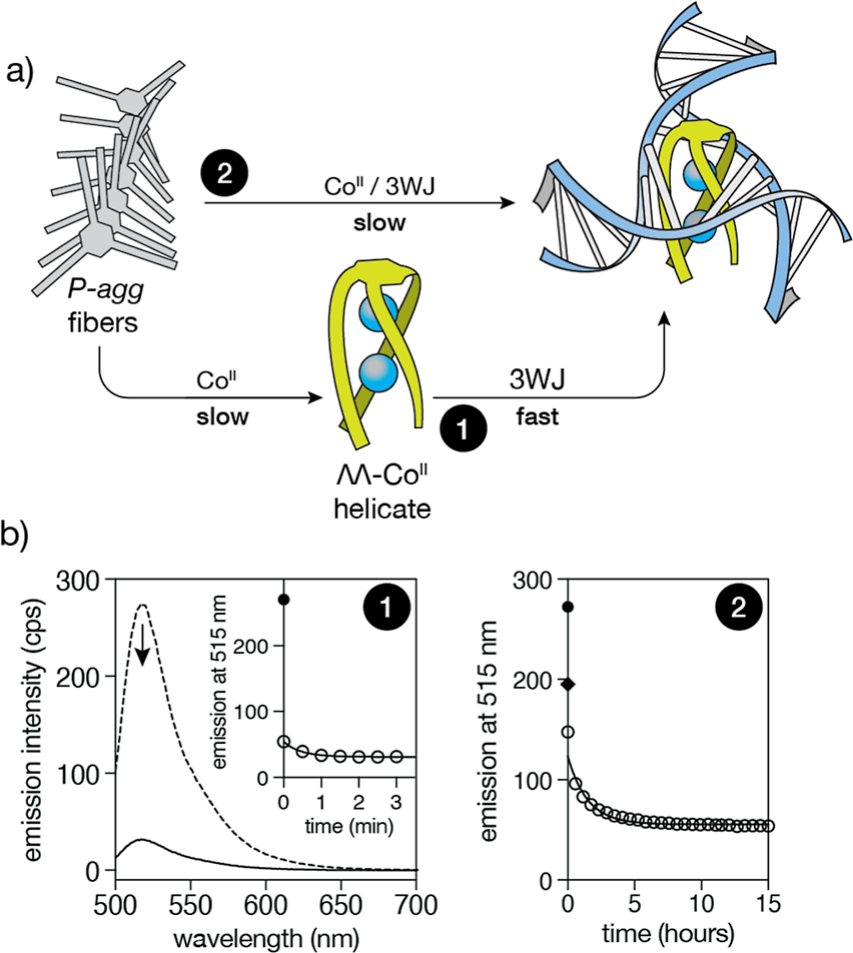
(a) Illustration of the 3WJ recognition processes in HEPES buffer from both (process 1) the preformed peptide helicate, ΛΛ-Co^II^_2_ **BTMA-1**, and (process 2) the chiral helical polymer (P-agg). (b) Left, fluorescence spectra of a 2 μM solution of 3WJ labeled with FAM (3WJ-FAM) in HEPES buffer (10 mM, 100 mM NaCl, pH 7.0) before (black circle) and after the addition of 3.75 eq of the preformed peptide helicate ΛΛ-Co^II^_2_ **BTMA-1** at time 0 and then every 30 s (black points). The fluorescence spectra stabilizes after c.a. Two minutes, indicating that the 3WJ recognition by the Co^II^ peptide helicate is complete. Right, fluorescence emission intensity at 515 nm of a 2 μM solution of 3WJ labeled with FAM (3WJ-FAM) in HEPES buffer (10 mM, 100 mM NaCl, pH 7.0) before (black circle) and after the addition of 3.75 eq of a dispersion of P-agg ([**BTMA-1**] = 100 μM) in HEPES buffer (P-agg) (black diamond) and after addition of 56.25 eq of Co^II^ ions at time 0 and then every 5 min (white circles). In this case, the emission intensity stabilizes after c.a. Ten hours, indicating that the 3WJ recognition by the Co^II^ peptide helicate is complete. λ_exc_ = 490 nm. 3WJ-FAM oligonucleotide sequences used in these experiments are described in the Supporting Information. Details of the experimental procedure are described in the main text.

All these findings demonstrate that supramolecular helical polymers can serve as dynamic precursors for discrete biomolecular recognition units, expanding their role beyond static architectures into bioresponsive materials. Therefore, this work not only provides a novel strategy for 3WJ targeting but also paves the way for the development of metal-responsive supramolecular therapeutics.

## Conclusion

In summary, here we demonstrate that a single molecule, **BTMA-1**, can adopt in physiological media two distinct chiral supramolecular architectures depending on the conditions: a chiral supramolecular helical polymer (*P*-agg) and a discrete chiral peptide helicate (ΛΛ-Co^II^_2_ **BTMA-1**) upon coordination with Co^II^ ions. Importantly, the Co^II^ helicate exhibits high affinity and selectivity for three-way DNA junctions (3WJs) over canonical dsDNA. Moreover, this peptide helicate can be oxidized in situ to generate the kinetically inert Co^III^ derivative, which also retains its selective 3WJ-binding properties.

Beyond these findings, we introduce a new paradigm in supramolecular chemistry by demonstrating that *P*-agg can act as an inert reservoir of **BTMA-1**, dynamically releasing bioactive ΛΛ-Co^II^_2_ **BTMA-1** helicates upon metal coordination in physiological conditions. This responsive supramolecular strategy enables spatiotemporal control of biomolecular recognition events, which could open the way for future applications in dynamic drug delivery. We strongly believe that this work highlights the unexplored potential of supramolecular helical polymers as precursors of discrete bioactive molecules, offering new opportunities in the interface of chemical biology and molecular materials.

## Supplementary Material



## Notes

Detailed author contributions; experimental procedures; peptide synthesis protocols; compound characterization (NMR and mass spectra); UV–vis, fluorescence, and ECD data; oligonucleotide sequences; data analysis; EMSA and TEM experiments; molecular modeling methods and optimized structures; as well as additional figures, tables, and references supporting the main text (PDF)
