# Supplementary material for: Unlocking a Biological Interface of Chiral Supramolecular Helical Polymers
Source: J Am Chem Soc. 2025 Jun 10;147(29):25254–63. doi: 10.1021/jacs.5c02902 (PMC12291456; doi:10.1021/jacs.5c02902)
Supplement: Supplementary file 1 [file ja5c02902_si_001.pdf]

Supporting Information

Unlocking a Biological Interface of Chiral Supramolecular Helical Polymers

Ana Alcalde-Ordóñez,[a]† Axel Sarmiento,[a]† Jacobo Gómez-González,[b] David Bouzada, [a] Manuel Núñez-Martínez,[b] Manuel Fernández-Míguez,[b] Rafael Rodríguez,[c] Félix Freire,*[b,c] M. Eugenio Vázquez*[b] and Miguel Vázquez López*[a]

---

[a] Prof. Miguel Vázquez López, Dr. Ana Alcalde-Ordóñez, Axel Sarmiento, Dr. David Bouzada
Centro Singular de Investigación en Química Biolóxica e Materiais Moleculares (CiQUS) and Departamento de Química Inorgánica
Universidade de Santiago de Compostela
Rúa Jenaro de la Fuente s/n, 15782 Santiago de Compostela, Spain
E-mail: miguel.vazquez.lopez@usc.es

[b] Prof. M. Eugenio Vázquez, Prof. Félix Freire, Dr. Jacobo Gómez-González, Dr. Manuel Núñez-Martínez, Manuel Fernández-Míguez
Centro Singular de Investigación en Química Biolóxica e Materiais Moleculares (CiQUS) and Departamento de Química Orgánica
Universidade de Santiago de Compostela
Rúa Jenaro de la Fuente s/n, 15782 Santiago de Compostela, Spain

[c] Prof. Félix Freire (current address), Dr. Rafael Rodríguez
CINBIO, Departamento de Química Orgánica
Universidade de Vigo
Campus Universitario Lagoas Marcosende, 36310 Vigo, Spain

† These authors contribute equal.

** To whom correspondence should be addressed: miguel.vazquez.lopez@usc.es*

Correspondence may also be addressed to eugenio.vazquez@usc.es and felixmanuel.freire@uvigo.gal

Author contributions

The following table describes the contribution of each author according to the CRediT taxonomy as described in A. Brand, L. Allen, M. Altman, M. Hlava, J. Scott, *Learn. Publ.* **2015**, *28*, 151–155. This taxonomy provides a detailed classification of the various roles performed by each author. The degree of contribution is coded as *lead* (black), *equal* (dark grey), or *supporting* (light grey).

	AAO	AS	JGG	DB	MNM	MFM	RR	FF	MEV	MVL
**Conceptualization.** Ideas, formulation of overarching research goals and aims.										
**Methodology.** Development or design of methodology; creation of models.										
**Validation.** Verification of the overall reproducibility of results and research outputs.										
**Formal Analysis.** Application of formal techniques to analyze the data.										
**Investigation:** helicate synthesis, metal binding, DNA binding studies.										
**Investigation:** electron microscopy studies, CD studies										
**Investigation:** molecular modelling studies										
**Resources.** Provision of reagents, materials, instrumentation, and analysis tools.										
**Data Curation.** Management to annotate, scrub and maintain research data.										
**Writing: Original Draft.** Preparation of the paper, specifically writing the initial draft.										
**Writing: Review & Editing.** Critical review, commentary or revision.										
**Visualization.** Preparation, of the paper, specifically visualization &/or presentation.										
**Supervision.** Oversight responsibility for the research planning & execution.										
**Project Administration.** Management and coordination of the research.										
**Funding Acquisition.** Acquisition of the financial support leading to this publication.										

1. Reagents

All the solvents employed for the synthesis of the Fmoc-βAla5Bpy-OH were obtained from different commercial sources: *Fisher Chemical* (acetonitrile for HPLC, chloroform, toluene, DMF, methanol, NaOH), *Scharlau* (absolute ethanol), *Sigma Aldrich* (5,5'-dimethyl-2,2'-bipyridine, $KMnO_4$, $SOCl_2$, hydrazine monohydrate, xylene, DIPEA), *Panreac* (celite and $NaNO_2$).

For the synthesis of the peptide ligand, the Fmoc-Arg(Pbf)-OH amino acid was provided by *Sigma Aldrich*. The resin employed for the SPPS was H-Rink-Amide *ChemMatrix* 35-100 mesh particle size from *Sigma Aldrich*. The 1,3,5-tris(bromomethyl)benzene was purchased from *Alfa Aesar*. Solvents for SPPS and organic synthesis were provided by *Fischer Scientific*.

The metal salt employed in the synthesis of the Co^{II} and Co^{III} metallopeptides, $Co(ClO_4)_2{\cdot}6H_2O$ and $(NH_4)_2Ce(NO_3)_6$, were provided by *Alfa Aesar*.

2. Experimental Methods

2.1. Mass spectrometry

Matrix-assisted laser desorption/ionization mass spectrometry (MALDI-MS) was performed with a *Bruker Autoflex MALDI-TOF* model in positive scan mode by direct irradiation of the matrix-adsorbed peptide. 4-HCCA (α-cyano-4-hydroxycinnamic acid) was the selected matrix for all these experiments.

2.2. UV-Vis Spectroscopy

UV measurements were made in a *Jasco V-630* spectrophotometer coupled to a *Jasco ETC-717* temperature controller, using a standard *Hellma* semi-micro cuvette 108.002-QS (10 mm light path). Measurements were made at 20 °C. Acquisition parameters were: 220-700 nm range, scan speed of 200 nm/min, resolution of 0.2 nm.

The experiments were performed at both 10 and 50 μM of BTMA-1. As a source of metal ions, $Co(ClO_4)_2{\cdot}6H_2O$ was used, while BTMA-1 was added from two stock solutions: 1 mM in DMSO and 10 mM

in HEPES buffer with 100 mM NaCl at pH 7.5. When working with DMSO as the sole solvent, spectral changes were observed immediately after Co^{II} addition. Measurements were taken for 10 minutes every minute. In contrast, when working with HEPES buffer, it was necessary to wait half an hour to observe the stabilization of the system. Measurements were taken for 30 minutes every 5 minutes.

2.3. Circular Dichroism

Electronic Circular Dichroism (ECD) measurements for solvent denaturation experiments were carried out using a *Jasco J-1500* spectropolarimeter. Spectra were recorded in the range of 550–260 nm with a data pitch of 1 nm. The CD scale was set to 200 mdeg/0.1 dOD, with a digital integration time (D.I.T.) of 1 second and a bandwidth of 1 nm. Measurements were acquired in continuous scanning mode at a speed of 500 nm/min, and each spectrum represents the average of two accumulations. The shutter was automatically controlled during acquisition. Baseline correction was applied by subtracting the spectrum of the corresponding solvent or blank (see Table S1).

**Parameter**	**Value**
Measurements range (nm)	550-260
Data pitch (nm)	1.00
CD scale	200 mdeg/0.1 dOD
D.I.T. (sec)	1.00
Bandwidth (nm)	1.00
Scanning mode	Continuous
Scanning Speed (nm/min)	500
Baseline Correction	Baseline
Shutter Control	Auto
Accumulations	2

Table S1. *Experimental parameters used for the ECD measurements performed on the Jasco J-1500.*

All other circular dichroism spectra were recorded using a *Jasco J-715* spectropolarimeter coupled to a *Neslab RTE-111* thermostated water bath, using a *Hellma* semi-micro cuvette 114F-10-40 (10 mm light pass). Scan speed was 200 nm/min and the obtained spectra are the mean of three accumulations (see Table S2). Metal-binding experiments were performed at both 10 and 50 μM of BTMA-1. As a source of metal ions, $Co(ClO_4)_2 \cdot 6H_2O$ salt was used while **BTMA-1** was added from two stock solutions at 1 mM in DMSO and 10 mM HEPES buffer with 100 mM NaCl at pH = 7.5. **BTMA-1-D** was added from a stock solution at 10 mM HEPES buffer with 100 mM NaCl at pH = 7.5. When working with DMSO as unique solvent, it was observed that the spectral changes occurred immediately after the addition of Co^{II}. Measurements were taken for 10 minutes every minute. In contrast, when working with HEPES buffer, it was necessary to wait half an hour to observe the stabilization of the system. Measurements were taken for 30 minutes every 5 minutes.

**Parameter**	**Value**
Measurements range (nm)	550-260
Data pitch (nm)	1.00
CD scale	200 mdeg/0.1 dOD
D.I.T. (sec)	1.00
Bandwidth (nm)	1.00
Scanning mode	Continuous
Scanning Speed (nm/min)	200
Baseline Correction	Baseline
Shutter Control	Auto
Accumulations	2

Table S2. *Experimental parameters used for the ECD measurements performed on the Jasco J-715.*

2.4. DNA hybridization

DNA oligonucleotides were obtained from *Biomers*. Concentration of the oligonucleotides was measured by UV-VIS by using the extinction coefficient given by the Commercial House *Biomers*. For the hybridization process, a stoichiometric mixture of the DNA strands was prepared in H_2O MQ and heated at 90°C for 10 minutes. After this time the mixture was slowly cooled down until room temperature, obtaining the resulting 3WJ/ds hybridized DNA.

Fluorescein-labelled 3WJ (3WJ-FAM):

Y1: `5′-FAM-TTTT CAC CGC TCT GGT CCT C-3′`
Y2: `5′-CAG GCT GTG AGC GGT G-3′`
Y3: `5′-GAG GAC CAA CAG CCT G-3′`

3WJ:

Y1: `5′-CAC CGC TCT GGT CCT C-3′`
Y2: `5′-CAG GCT GTG AGC GGT G-3′`
Y3: `5′-GAG GAC CAA CAG CCT G-3′`

Fluorescein-labelled dsDNA (dsDNA-FAM):

Z1: `5′-FAM-TTTT AAC ACA TGC AGG ACG GCG CTT-3′`
Z2: `5′-AAG CGC CGT CCT GCA TGT GTT-3′`

B-DNA:

Z1: `5′-AAC ACA TGC AGG ACG GCG CTT-3′`
Z2: `5′-AAG CGC CGT CCT GCA TGT GTT-3′`

2.5. Synthesis of BTMA-1 and BTMA-1-D

2.5.1. Solid Phase Peptide Synthesis

BTMA-1 and **BTMA-1-D** were synthesized using standard Fmoc-based solid-phase peptide synthesis protocols on a 0.1 mmol scale, employing H-Rink amide ChemMatrix resin (loading: 0.5 mmol/g, 35–100 mesh). Amino acid couplings were performed manually using HATU as the coupling reagent. Each amino acid was pre-activated for 1 min in 0.2 M DIEA/DMF (4 mL) prior to addition to the resin, and couplings were allowed to proceed for 60 min. A 5-fold excess of L-Arg (for **BTMA-1**) or D-Arg (for **BTMA-1-D**) was used, while Fmoc-βAla5Bpy-OH was coupled in 3.5-fold excess. Final Fmoc deprotection was carried out with 20% piperidine in DMF for 20 min. The key step in the synthesis was the on-resin S_N2 coupling of the trivalent benzene core. For this, the resin was suspended in a solution of 1,3,5-tris(bromomethyl)benzene (0.33 eq.) and DIEA (3 eq.) in 5 mL of DMF and stirred at room temperature for 24 h. The resin was then filtered and washed thoroughly with DMF (3×).

2.5.2. Cleavage from the resin and final deprotection

Cleavage and deprotection of the peptide ligands were simultaneously performed by treatment of the resin-bound peptide for 2.5 h with an acidic cocktail containing 50 µL of CH_2Cl_2, 25 µL of H_2O, 25 µL of TIS (triisopropylsilane), and 900 µL of TFA (1 mL of cocktail / 40 mg of resin). The resin was filtered, and the TFA filtrate was concentrated under a nitrogen stream to an approximate volume of 1 mL, and then added onto ice-cold diethyl ether (20 mL). After 10-30 min, the precipitate was centrifuged and washed again with 5 mL of ice-cold ether. The solid residues were dried under N_2, re-dissolved in a mixture of H_2O and CH_3CN and purified by preparative HPLC for the obtention of the pure compounds.

2.6. HPLC and UHPLC

2.6.1. UHPLC-MS

Peptide analysis was performed by analytical UHPLC-MS with an *Agilent 1200 series LC/MS* using a *SB C18* (1.8 *µ*m, 2.1 × 50mm) analytical column from *Phenomenex*. Standard conditions for analytical UHPLC consisted of a linear gradient from 5% to 95% of solvent B in 12 min at a flow rate of 0.350 mL/min (A: water 0.1% TFA, B: acetonitrile 0.1% TFA). The compound was detected by UV absorption at 310 nm. Electrospray Ionization Mass Spectrometry (ESI/MS) was performed with an *Agilent 6120 Quadrupole LC/MS* model in positive scan mode using direct injection of the purified peptide solution into the MS detector.

2.6.2. Preparative HPLC

Peptide purification was performed by preparative RP-HPLC with a *Waters 1500 series Liquid Chromatograph* using a *Sunfire Prep C18 OBD* (5 µm, 19 × 150 mm) reverse-phase column from *Waters*. Standard conditions for preparative RP-HPLC consisted of an isocratic regime during the first 5 min, followed by linear gradient of 10-40% of B in 30 min (A: water 0.1% TFA, B: acetonitrile 0.1% TFA). Collected fractions with pure products were lyophilized with a *ThermoSavant Modulyo D lyophilizator* equipped with an *Edwars RV* high vacuum pum.

2.7. Fluorescence spectroscopy

Metal binding studies were performed at a fixed concentration of 50 μM of **BTMA-1** in an Edinburg Fluoracle FS5, employing temperature control cell holder SC-26 fixed to 20ºC. All measurements were made with a Hellma semi-micro cuvette (108F-QS).

DNA binding studies were performed using a *Varian Cary Eclipse* Fluorescence Spectrophotometer coupled to a *Cary* Single *Cell Peltier* temperature controller (*Agilent Technologies*). All measurements were made with a *Hellma* semi-micro cuvette 114F-10-40 (10 mm light pass) at 20 °C.

2.7.1. Metal binding studies

In the case of the solvent DMSO, no kinetic inertia was observed after the addition of the metal ion aliquots during titrations. In contrast, in aqueous solution, two types of experiments are performed: with and without overnight incubation. As a source of metal ions, $Co(ClO_4)_2·6H_2O$ salt was used while **BTMA-1** was added from two stock solutions at 115ºC for at 1 mM in DMSO and 10 mM HEPES buffer with 100 mM NaCl at pH = 7.5. Dissociation constants were calculated using *GraphPad Prism 10* software.

2.7.2. DNA binding studies

The fluorescence emission of a 2 µM solution of the fluorescein-labelled 3WJ/dsDNA (3WJ-FAM/dsDNA-FAM) in HEPES buffer (10 mM, 100 mM NaCl, pH 7.0) was monitored after the addition of increasing amounts of the preformed ΛΛ-Co^{II}_2**BTMA-1** or ΛΛ-Co^{III}_2**BTMA-1** (λ_{exc} = 490 nm). Dissociation constants were calculated using *Dynafit 4.0* software.

2.7.3. DNA binding kinetic studies

The fluorescence emission of a 2 µM solution of the fluorescein-labelled 3WJ in HEPES buffer (10 mM, 100 mM NaCl, pH 7.0) was monitored at 515 nm with time after the addition of either 3.75 equivalents of the preformed ΛΛ-Co^{II}_2**BTMA-1** and 3.75 equivalents of *P*-agg in the presence of Co^{II} ions (15 equivalents regarding to **BTMA-1**). λ_{exc} = 490 nm.

2.7.4. Data analysis with Dynafit 4.0

Fluorescence DNA-binding titration data were analyzed using the *DynaFit 4.0* software, which characterizes the reacting system in terms of stoichiometric equations instead of mathematical notation; the chemical equations are then translated into the underlying mathematical equations using matrix theory. [*Ref. 31 in the manuscript*] *DynaFit* is available free of charge at http://www.biokin.com/dynafit/. The program requires plain text files, known as scripts, that contain the chemical model underlying the experimental data, the values of model parameters, such as starting concentrations of reactants, as well as information about location of the files. A typical script used in the analysis of these type of titrations is included below. The file has been commented to indicate the purpose of the keywords and sections, but the reader is recommended to review the *DynaFit* scripting manual distributed along the program or available at the *DynaFit* website.

```
[task]                          ;semicolons indicate comments
 task = fit                     ;nature of the calculation to be performed
 data = equilibria
[mechanism]                     ;Free-form 1:1 binding model with Kd
 R + L <==> RL : Kd dissoc;to be calculated as dissociation constant
[constants]                     ;Initial Kd value for iteration
 Kd = 1.0 ?                     ;the "?" indicates that this will be optimized
[concentrations]
 R = 2.0                        ;Fixed conc. of the DNA during the peptide titration
[responses]                     ;contribution to the spectroscopic signal of each
 R = 0.1 ?                      ;of the different components of the equilibrium
 RL = 1.5 ?                     ;these will be optimized ("?" after the values)
[data]                          ;location of files and information about the data
 variable  L                    ;the species that changes conc. during the titration
 offset auto ?
 directory ./exp/SH             ;file path (relative to DynaFit program location)
```

```
 extension txt
 file f1                ;name of the experimental data file
[output]
 directory ./exp/SH/out   ;path indicating location of DynaFit output files
[settings]              ;cosmetic settings that control DynaFit graphics
 {Output}               ;fits were exported & finally plotted with
 XAxisUnit = uM         ;GraphPad Prism 7.0c. GraphPad Software,
 BlackBackground = n    ;La Jolla California, www.graphpad.com
 XAxisLabel = [peptide]
 YAxisLabel = anisotropy
 WriteTXT = y
```

2.8. Polyacrylamide gel electrophoretic analysis

PAGE experiments with non-labellled DNA (3WJ/dsDNA) were performed with a *Bio-Rad Mini Protean* gel system, powered by an electrophoresis power supplies *PowerPac Basic model*, maximum power 150 V, frequency 50–60 Hz at 130 V (constant V).

The DNA sequences used in these studies can be found in section 2.4 of this file. Binding reactions were performed for 30 min in 18 mM Tris-HCl (pH 7.5), 90 mM KCl, 1.8 mM $MgCl_2$, 9% glycerol, 0.11 mg/mL BSA, and 2.2% NP-40. For the experiments we used 200 nM of 3WJ and 200 nM of dsDNA with the Co^{II} metallopeptide or 50 nM of dsDNA with the Co^{III} metallopeptide, and different quantities of the preformed ΛΛ-Co^{II}_2**BTMA-1** or ΛΛ-Co^{III}_2**BTMA-1** in a total incubation volume of 20 µL. After incubation for 30 min at room temperature, products were resolved by PAGE using a 10% non-denaturing polyacrylamide gel and 0.5 × TBE buffer (0.445 M Tris, 0.445 M boric acid) for 35 min at 130 V at 25 °C, and analyzed by staining with SyBrGold (Molecular Probes: 5 µL in 50 mL of 1 × TBE) for 10 min, followed by fluorescence visualization (*BioRad* GelDoc XR+ molecular imager).

2.9. TEM/STEM studies

STEM measurements were performed on a *LEO-435VP electron microscope* equipped with an energy dispersive X-ray (EDX) spectrometer. To study the morphology of the aggregates, dispersions of **BTMA-1** at a concentration of 0.1 mg/mL in DMSO and HEPES buffer in the presence and absence of Co^{II} ions were drop-casted onto carbon film supported copper grids and allowed to dry at rt for 12 h.

2.10. Molecular modeling studies

All **BTMA-1** peptide helicates were built from scratch using *Avogadro*. Geometries and harmonic frequencies for all non-standard residues were calculated with *Gaussian 16*, employing the B3LYP functional with Grimme's D3 empirical dispersion correction. Cobalt atoms were described using the Stuttgart–Dresden (SDD) effective core potential and basis set, while C, N, O, and H atoms were treated with the 6-311+G(d,p) basis set. Atomic partial charges were derived using the Restrained Electrostatic Potential (RESP) model.

Molecular dynamics (MD) simulations were performed in *AMBER*. Each system was solvated in a box of pre-equilibrated TIP3P water molecules, and counterions (Cl^-) were added to neutralize the total charge. For simulations in DMSO, the solvent parameters were taken from the Bryce Group repository (University of Manchester) using the *DMSO.off* and *DMSO.frcmod* files.[1]

Standard amino acids were parameterized using the AMBER ff19SB force field, whereas the remaining atoms were described using the General AMBER Force Field (GAFF). The Co–bipyridine coordination environment was modeled using force constants and equilibrium parameters obtained through the *MCPB.py* module employing the Seminario method.

The MD protocol began with a 3000-step minimization of the solvent, followed by system equilibration. Solvent molecules were first heated to 300 K over 2 ns, then equilibrated under constant pressure (1 bar) using periodic boundary conditions. Restraints on the system were progressively removed during subsequent equilibration stages.

Once fully equilibrated, the systems were subjected to 1 µs production MD simulations to explore their conformational landscapes. The final **BTMA-1** peptide helicate structures correspond to the most populated conformational cluster, with representative frames extracted from the simulation trajectory.

2.11. NMR studies

NMR samples of **BTMA-1** were prepared at a concentration of 1 mM in DMSO-d_6 and in 90% H_2O/10% D_2O, respectively. All spectra were recorded on a Bruker NEO 750 MHz spectrometer equipped with a z-axis

1 Fox, T.; Kollman, P. Application of the RESP Methodology in the Parametrization of Organic Solvents. Journal of Physical Chemistry B 1998, 102, 8070–8079.

pulsed-field gradient triple-resonance PA-TXI-HFCN probe (^{1}H, ^{13}C, ^{15}N), using Norell® Selected Series 3 mm glass tubes. One-dimensional (1D) ^{1}H spectra were acquired over a temperature range of 298–333 K in DMSO-d$_6$. For measurements in H_2O/D_2O, water suppression was applied via pre-saturation, and ^{1}H spectra were recorded from 278 K to 333 K in H_2O/D_2O and from 298 K to 333 K in DMSO-d$_6$.

3. Experimental data

3.1. Synthesis of Fmoc-βAla5Bpy-OH (1)

The synthesis of this chelating residue was carried out following the methodology published by our group [*ref. 11 in the manuscript*]. All the obtained products were analyzed by HPLC-MS to test their purity.

3.1.1. Synthesis of 2,2'-bipyridine-5,5'-dicarboxilic acid (2)

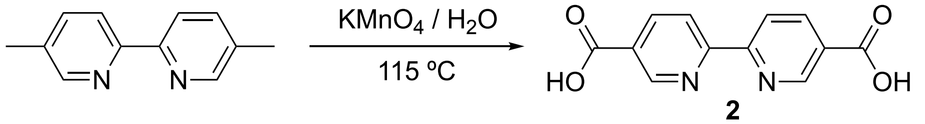

2

A mixture of 39 g of potassium permanganate and 7 g of 5,5'-dimethyl-2,2' -bipyridine in 250 mL of H_2O was heated at 115ºC for 2 h, cooled at room temperature, and filtered through Celite. The filtrate was cooled to 4 ºC and acidified with HCl until precipitation of a white solid, which was filtered, washed with water and lyophilized to afford the desired product in a 93% yield (8.7 g)

^{1}H-NMR (500 MHz, DMSO-*d*$_6$, δ): 13.50 (br); 9.18 (dd, ^{4}J = 2.15, ^{5}J = 0.8 Hz 2H); 8.55 (dd, ^{3}J= 8.3; ^{5}J = 0.8 Hz, 2H); 8.44 (dd, ^{3}J = 8.3; ^{4}J= 2.15 Hz, 2H).

^{13}C-NMR (125 MHz, DMSO-*d*$_6$, δ): 165.49, 156.84, 149.82, 137.97, 126.65, 120.62.

MALDI-TOF (m/z) $[M+H]^+$ calculated for $[C_{12}H_8N_2O_4]$ 245.0; found 245.0.

3.1.2. Synthesis of diethyl [2,2'-bipyridine]-5,5'-dicarboxylate (3)

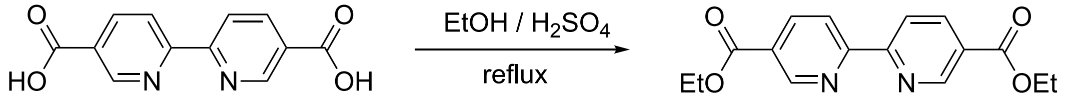

2 **3**

2,2'-bipyridine-5,5'-dicarboxylic acid (10.0 g, 41 mmol) was suspended in 150 mL of absolute ethanol. Concentrated sulfuric acid (20.0 mL) was slowly added, and the resulting mixture was refluxed for 18 h. The solution was cooled at room temperature and added over 400 mL of water at 4 ºC causing the precipitation of a white solid, which was filtered, washed with water and lyophilized. 11.5 g (93.0 %)

^{1}H-NMR (500 MHz, DMSO-*d*$_6$, δ): 9.20 (dd, ^{4}J = 2.15, ^{5}J = 0.8 Hz 2H); 8.57 (dd, ^{3}J= 8.3; ^{5}J = 0.8 Hz, 2H); 8.46 (dd, ^{3}J = 8.3; ^{4}J = 2.15 Hz, 2H); 4.0 (q, ^{3}J = 7.1 Hz, 4H); 1.37 (t, ^{3}J = 7.1 Hz, 6H).

^{13}C-NMR (125 MHz, DMSO-*d*$_6$, δ): 164.2, 157.2, 149.8, 138.0, 126.2, 121.0, 61.1, 13.9.

MALDI-TOF (m/z) $[M+H]^+$ calculated for $[C_{16}H_{16}N_2O_4]$ 301.1; found 301.1.

3.1.3. Synthesis of Ethyl 5'-Carbohydrazido-2,2'-bipyridine-5-carboxylate (4)

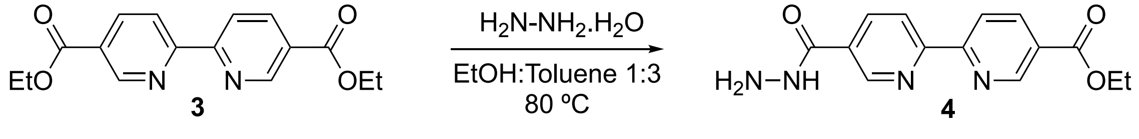

3 **4**

A mixture of diethyl 2,2'-bipyridine-5,5'-dicarboxylate (15.00 g, 50 mmol) and hydrazine hydrate (3.75 mL, 55 mmol) in a solution of EtOH (42 mL) and toluene (128 mL) was heated at 80 °C for 48 h. The precipitate was filtered, washed with $CHCl_3$ and dried under vacuum 11.4 g (80%). The unreacted diethyl ester was concentrated and mixed again to obtain a global yield of (90%).

^{1}H-NMR (500 MHz, DMSO-*d*$_6$, δ): 10.1 (br, 1H), 9.2 (d, ^{4}J = 2 Hz, 1H), 9.1 (d, ^{4}J = 2 Hz, 1H), 8.57 (d, ^{3}J = 8.4 Hz, 1H), 8.52 (d, ^{3}J = 8.4 Hz, 1H), 8.45 (dd, ^{3}J = 8.4 Hz, ^{4}J = 2 Hz, 1H), 8.35 (dd, ^{3}J = 8.4 Hz, ^{4}J = 2 Hz, 1H), 4.37 (q, 2H), 1.35 (t, 3H).

^{13}C-NMR (125 MHz, DMSO-*d*$_6$,δ): 164.5, 163.8, 157.8, 155.8, 150.0, 148.1, 138.2, 136.2, 129.6, 126.1, 120.9, 61.3, 14.1.

MALDI-TOF (m/z) $[M+H]^+$ calculated for $[C_{14}H_{14}N_4O_3]$ 287.1; found 287.1.

3.1.4. Synthesis of Ethyl 5'-Carbohydrazido-2,2'-bipyridine-5-carboxylate (5)

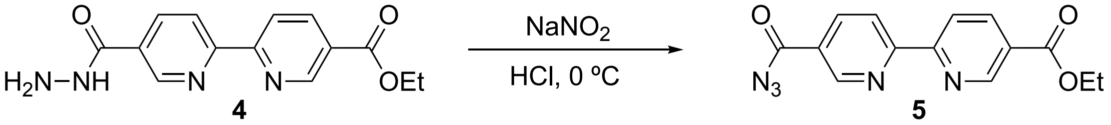

A stirred solution of 5-(ethoxycarbonyl)-5'-carbohydrazido-2,2'-bipyridine (5.7 g, 20 mmol) in concentrated HCl (100 mL) was cooled to 0 °C, and then an aqueous solution of $NaNO_2$ (1.73 g, 25 mmol; 15 mL) was added dropwise, maintaining the temperature at 0 °C. After 60 min, the yellow solution was diluted with water (300 mL) to precipitate the monoester 5 as a white powder, which was filtered, washed with water, and lyophilized 5.5 g (92 %)

^{1}H-NMR (500 MHz, Acetone-d^6,δ): 9.13 (d, ^4J = 1.91 Hz, 1H), 9.10 (d, ^4J = 1.91 Hz, 1H), 8.71 (d, ^3J = 12.5 Hz, 1H), 8.69 (d, ^3J = 12.5 Hz, 1H), 8.517 (m, 2H), 4.31 (q, ^3J = 7.15 Hz, 2H), 1.29 (t, ^3J= 7.15, 3H).

^{13}C-NMR (125 MHz, Acetone-d_6,δ): 170.8, 164.5, 159.2, 157.7, 150.3, 150.0, 138.0, 137.9, 127.0, 121.3, 61.2, 13.6.

MALDI-TOF (m/z) $[M+H]^+$ calculated for $[C_{14}H_{11}N_5O_3]$ 298.1; found 298.1

3.1.5. Synthesis of Ethyl 5'-(ethoxycarbonyl)amino-2,2'-bipyridine-5-carboxylate (6)

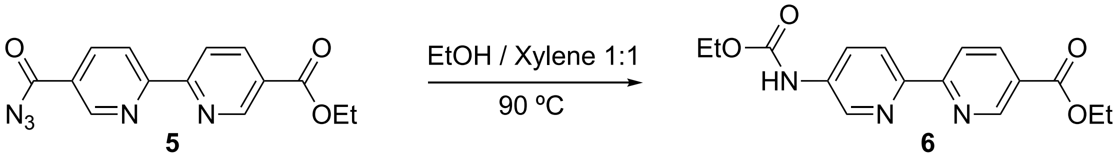

A solution of ethyl 5'-carbazido-2,2'-bipyridine-5-carboxylate (8.6 g, 29 mmol) in a mixture of EtOH (100 mL) and xylene (100 mL) was heated at 90 °C for 4 h. The solvent was evaporated under reduced presure, and the yellow residue was washed with EtOH and dried in vacuo. 8.21 g (90%).

^{1}H-NMR (500 MHz, DMSO-d_6, δ): 10.13 (s, 1H), 9.12 (d, ^4J = 2 Hz, ^5J = 1 Hz, 1H), 8.70 (d, ^4J = 2 Hz, 1H), 8.45 (d, ^3J = 8 Hz, 1H), 8.40 (dd, ^3J = 8.3Hz, ^5J = 1 Hz, 1H), 8.35 (dd, ^3J = 8.3 Hz, ^4J = 2 Hz, 1H), 8.08 (dd, ^3J = 8 Hz, ^4J = 2 Hz, 1H), 4.36 (q, ^3J = 7.2Hz, 2H), 4.17 (q, ^3J = 7.2 Hz, 2H), 1.35 (t, ^3J = 7.2 Hz, 3H), 1.27 (t, ^3J = 7.2 Hz, 3H).

^{13}C-NMR (125 MHz, DMSO-d_6, δ): 164.17, 158.07, 153.06, 149.40, 147.44, 139.05, 137.42, 136.77, 124.96, 124.46, 121.21, 119.11, 60.66, 60.32, 13.95, 13.65.

MALDI-TOF (m/z) $[M+H]^+$ calculated for $[C_{16}H_{17}N_3O_4]$ 316.1; found 316.1.

3.1.6. Synthesis of 5'-Amino-2,2'-bipyridine-5-carboxylic acid hydrochloride (7)

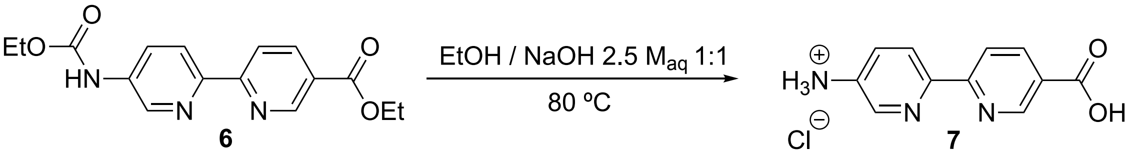

A stirred solution of ethyl 5'-[(ethoxycarbonyl)amino]-2,2'-bipyridine-5-carboxylate (14.55 g, 45.9 mmol) in a mixture of EtOH (50 mL) and 2.5 M aqueous NaOH (50 mL) was heated at 75 °C for 14 h. The EtOH was concentrated in vacuum, and the aqueous solution was acidified with HCl to afford a bright yellow precipitate, which was filtered, washed with cold water, and lyophilized 9.80 g (85%).

^{1}H-NMR (500 MHz, D_2O-d_2, δ): 8.65 (d, ^4J = 2.8 Hz, 1H), 7.98 (dd, ^3J = 8.5 Hz, ^4J = 2.9 Hz, 1H), 7.81 (d, ^4J = 2.9 Hz, 1H), 7.55, 7.61 (d, ^3J = 8.5 Hz, 2H), 7.00 (dd, ^3J = 8.5 Hz, ^4J = 2.8 Hz, 1H).

^{13}C-NMR (125 MHz, D_2O-d_2, δ): 176.04, 159.6, 152.4, 147.5, 147.4, 141.34, 139.8, 133.8, 126.7, 126.2, 123.3.

MALDI-TOF (m/z) $[M+H]^+$ calculated for $[C_{11}H_9N_3O_2]$ 216.1; found 216.1

3.1.7. Synthesis of Fmoc-βAla5Bpy-OH (1)

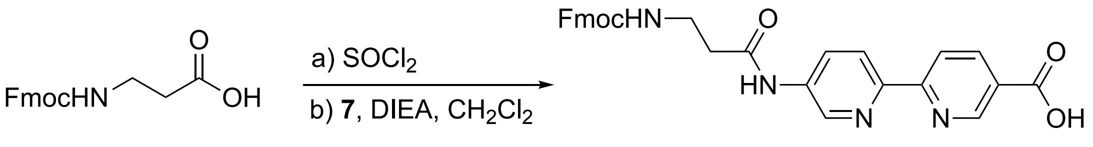

Fmoc-βAla5Bpy-OH, **1**

Over 4.58 g (14.72 mmol) of Fmoc-βAla 10 mL of $SOCl_2$ was added carefully at room temperature. The solution was stirred magnetically for 30 min, the thionyl chloride was evaporated in vacuum and the yellow solid was washed with CH_2Cl_2 (3x10 mL), and dried under reduced pressure. Compound **7** (3.7 g, 14.8 mmol), 20 mL of CH_2Cl_2 and 5 mL of DIEA were added. The suspension was stirred at room temperature overnight. The solvent was evaporated, the solid was suspended in acetonitrile 0.1 %TFA/H2O (2:1), centrifuged and washed with acetonitrile and H_2O and lyophilized 6.70 g (89 %).

Regarding the nomenclature of the coordinating residue **1**, Fmoc is the amine protecting group necessary to introduce the 2,2'-bipyridine residue into a peptide sequence by SPPS methodology. βAla is the amino acid attached to the amino group of the 2,2'-bipyridine amino acid. This extra amino acid is key to being able to integrate the 2,2'-bipyridine amino acid into the peptide sequence, as its amino group is not basic enough to form peptide bonds by a SPPS methodology. The number 5 indicates that the substitutions of the 2,2'-bipyridine molecule to transform it into an artificial amino acid have been made at their 5 and 5' positions.

^{1}H–RMN (300 MHz, DMSO-*d*6, δ): 10.44 (s, 1H); 9.13 (d, ^4J = 1.36 Hz, 1H); 8.89 (d, ^4J = 2.12 Hz, 1H); 8.42 (m, 3H); 8.25(dd, ^3J = 8.48 Hz, ^4J = 2.12 Hz, 1H); 7.88 (d, ^3J = 7.46 Hz, 2H); 7.67 (d, ^3J = 7.38 Hz, 2H); 7.49 (t, ^3J = 5.34 Hz, NH); 7.40 (t, ^3J = 7.21 Hz, 2H); 7.30 (t, ^3J = 7.38 Hz, 2H); 4.30 (d, ^3J = 6.95 Hz, 2H); 4.21 (t, ^3J = 6.95 Hz, 1H) 3.32 (t, ^3J = 6.70 Hz, 2H); 2.60 (t, ^3J = 6.70 Hz, 2H).

^{13}C-NMR (75 MHz, DMSO-*d*6, δ): 169.76, 168.62, 157.23, 150.94, 149.21, 144.74, 143.47, 142.27, 141.59, 140.28, 138.29, 138.08, 129.73, 128.09, 125.96, 122.14, 120.87, 120.79, 118.73, 110.34, 67.38, 63.77, 47.62, 41.71.

MALDI-TOF (m/z) $[M+H]^+$ calculated for $[C_{29}H_{25}N_4O_5]$ 509.1; found 509.1.

3.2. Synthesis and characterization of the peptide ligands

*3.2.1. **BTMA-1***

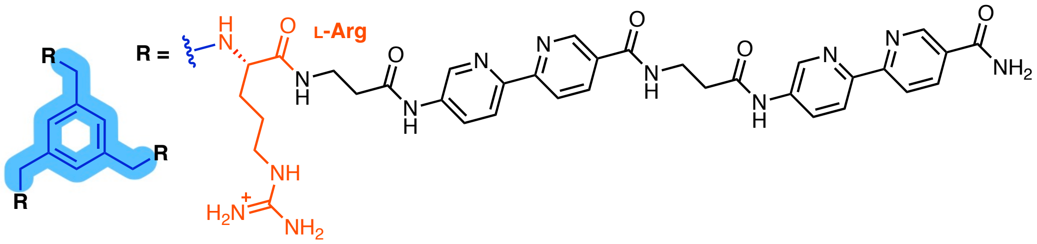

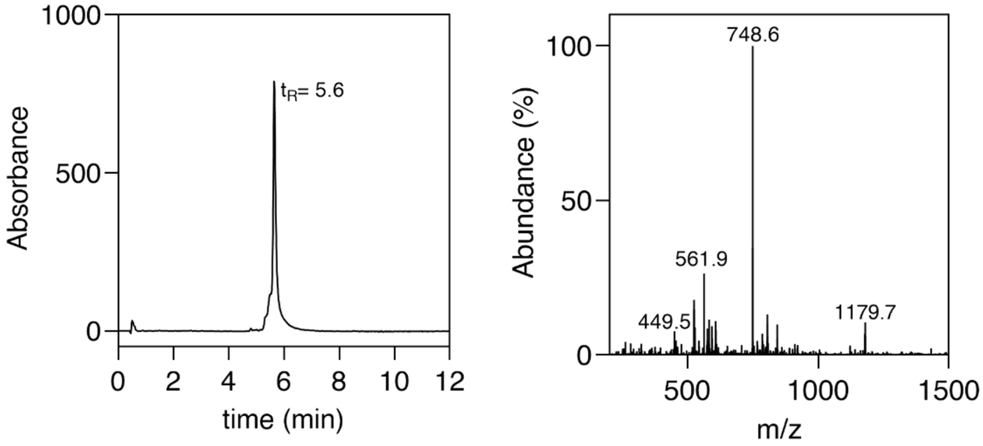

***Figure S1.** Left: chromatogram of the purified **BTMA-1**. Right: ESI mass spectra corresponding to the peak with t_R of 5.6 min.*

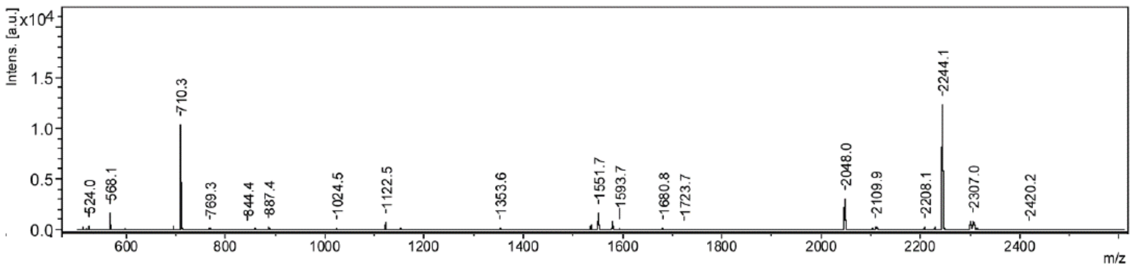

Figure S2. *MALDI spectra of* ***BTMA-1****.*

HPLC-MS (ESI): (5-95% B, t_R= 5.6 min) Calculated for $C_{111}H_{123}N_{39}O_{15}$ = 2242.01; found $[M+2H+TFAH]^{2+}$ = 1179.7; $[M+3H]^{3+}$ = 748.6; $[M+4H]^{4+}$ = 561.9; $[M+5H]^{5+}$ = 449.5.

MS (MALDI-TOF): Calculated for $C_{111}H_{123}N_{39}O_{15}$ = 2242.01; found: $[M+H]^{+}$ = 2244.1.

3.2.2. ***BTMA-1-D***

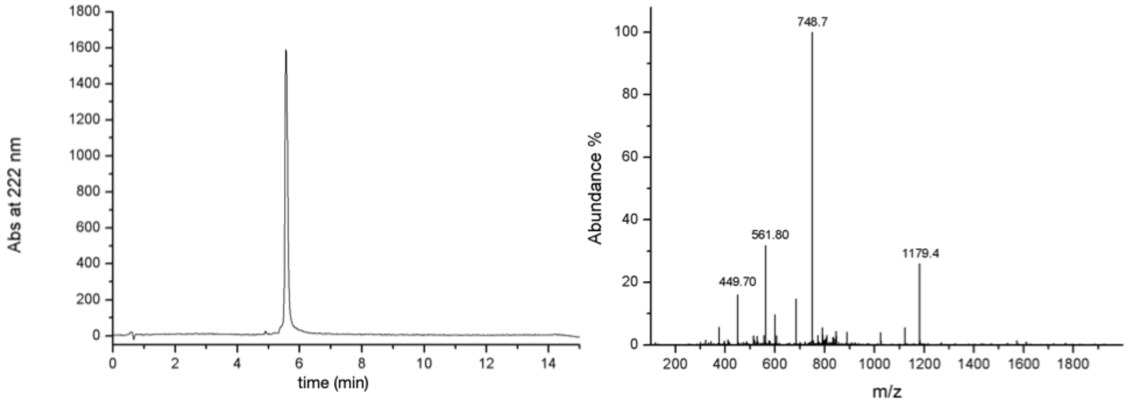

Figure S3. *Left: chromatogram of the purified* ***BTMA-1-D****. Right: ESI mass spectra corresponding to the peak with t_R of 5.6 min.*

3.3. Synthesis of the discrete ΛΛ/ΔΔ-Co^{II} peptide helicates

3.3.1. Fluorescence titration in HEPES buffer (overnight preincubation of the samples)

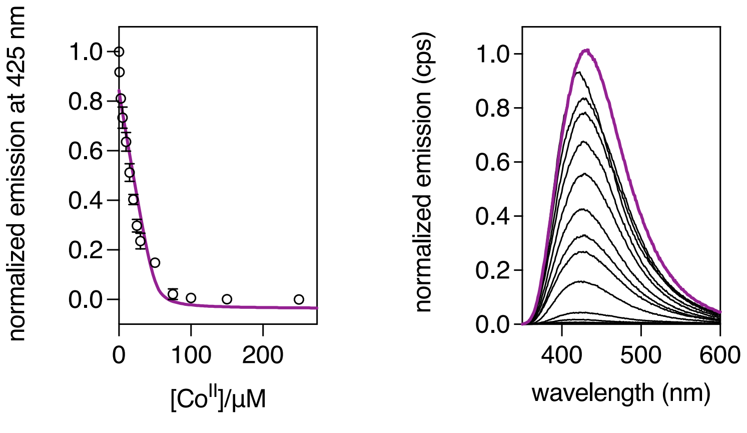

Figure S4. *Right, fluorescence titration experiment of a dispersion of P-agg ([**BTMA-1**] = 50 µM) in HEPES buffer (10 mM, 100 mM NaCl, pH 7.0) with increasing amounts of Co^{II} ions (black lines of decreasing intensity). The samples containing different ratios of Co^{II} and P-agg were incubated overnight before performing the corresponding fluorescence spectra. Left, titration profile at λ_{em}= 425 nm of three independent experiments and the best fit according to a 1:1 (L:M_2) binding model in Prism (violet line). The dissociation constant calculated was 0.9 ± 0.6 µM. λ_{ex}= 305 nm.*

3.3.2. ECD studies in HEPES buffer

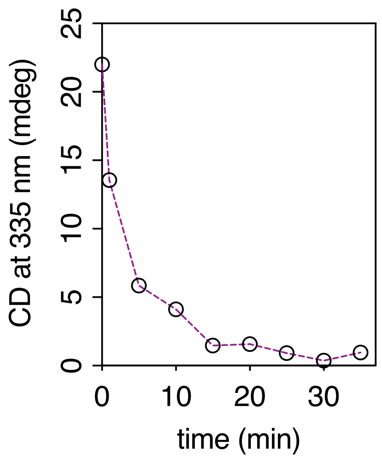

Figure S5. *ECD titration profile at 335 nm of the experiment describe in Figure 3b (down) of the manuscript. Experiment performed in HEPES buffer (10 mM, 100 mM NaCl, pH 7.0). Concentration of* ***BTMA-1*** *in the P-agg dispersion: 50 µM. Equivalents of Co^{II}: 15. Further details of the experimental procedure are described in the main text and in Sections 2.2 and 2.3 of this file.*

3.3.3. UV-vis and ECD studies in DMSO and HEPES buffer

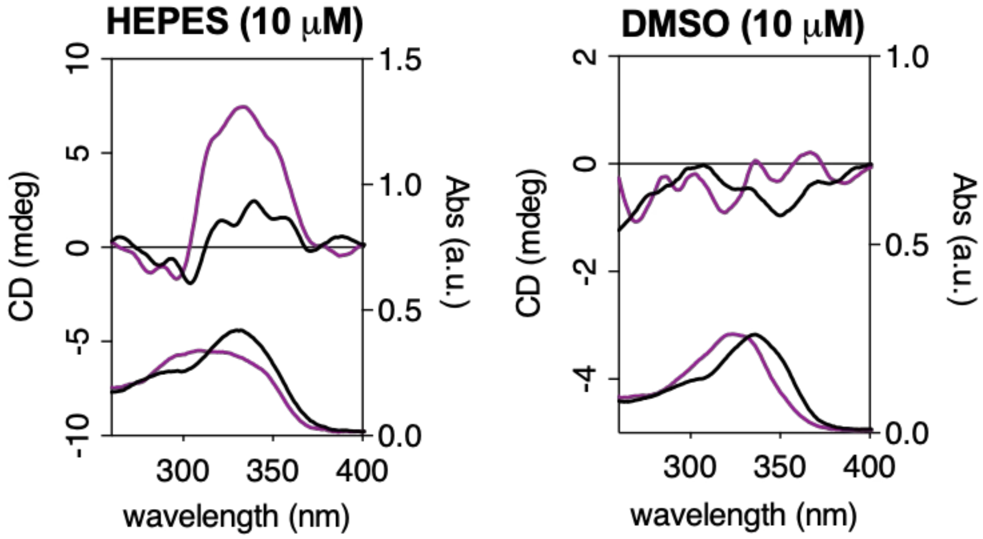

Figure S6. *Left, evolution with time of the ECD (up) and UV-vis (bottom) spectra of a dispersion of P-agg ([***BTMA-1***] = 10 µM) in the presence of 15 eq. of Co^{II} ions in HEPES buffer (10 mM, 100 mM NaCl, pH 7.0). The violet line corresponds to the initial state and the black line to the final state. The spectral changes ended c.a. 15 minutes after the start of the experiments. Right, evolution with time of the ECD (up) and UV-vis (bottom) spectra of a 10 µM solution of* ***BTMA-1*** *in the presence of 15 eq. of Co^{II} ions in DMSO. The violet line corresponds to the initial state and the black line to the final state. In this case the spectral changes observed were immediate. Further details of the experimental procedure are described in the main text and in Sections 2.2 and 2.3 of this file.*

3.3.4. Solvent-induced reversibility of helicity in Co^{II} peptide helicates

To investigate the reversibility of the solvent-induced chiral inversion observed for the Co^{II} peptide helicates, we prepared two stock solutions (1 mM) of **BTMA-1** with 15 equivalents of Co^{II} ions, one in HEPES buffer (10 mM HEPES, 100 mM NaCl, pH 7.0) and the other in pure DMSO. From these, four final samples were prepared at a concentration of 50 µM **BTMA-1** in:

(i) pure HEPES buffer,

(ii) pure DMSO,

(iii) 95% HEPES buffer / 5% DMSO, and

(iv) 95% DMSO / 5% HEPES buffer.

Electronic Circular Dichroism (ECD) spectra were recorded immediately after sample preparation. The results obtained clearly show that the Cotton effect is reversed when the helicate is transferred from HEPES buffer to DMSO and vice versa, confirming the complete reversibility of the solvent-controlled helicity switch. These findings provide strong experimental support for the dynamic nature of the folding process and the key role of solvent environment in modulating the chiral configuration of the metal complex.

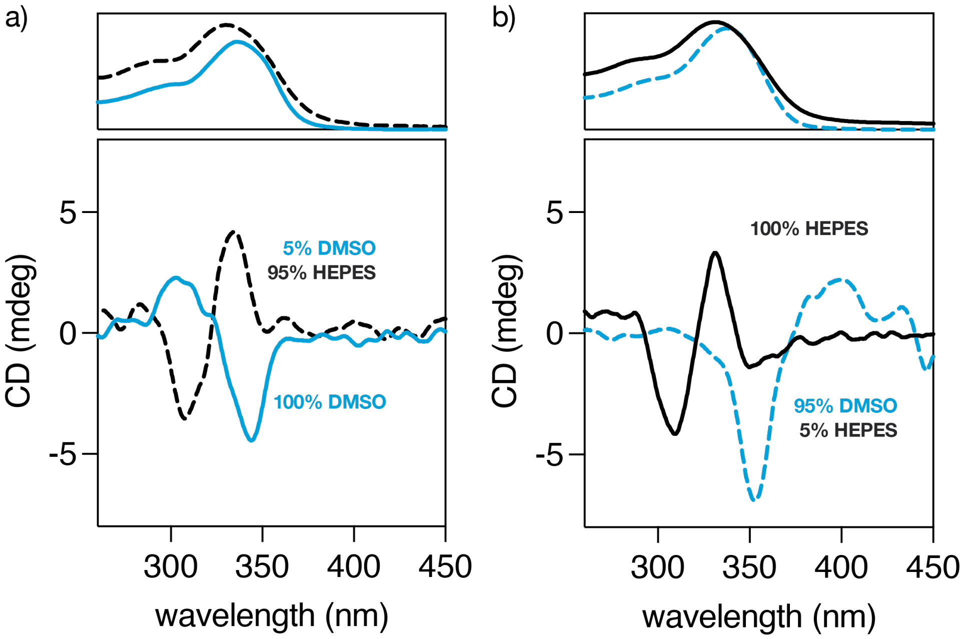

Figure S7. *a) UV–vis (top) and ECD (bottom) spectra of the Co^{II} peptide helicate derived from* ***BTMA-1*** *in pure DMSO (black dashed lines) and in a 95% HEPES buffer / 5% DMSO mixture (blue solid lines).(b) UV–vis (top) and ECD (bottom) spectra of the Co^{II} peptide helicate in pure HEPES buffer (black solid lines) and in a 95% DMSO / 5% HEPES mixture (blue dashed lines). In all samples, the concentration of* ***BTMA-1*** *is 50 µM and 15 equivalents of Co^{II} ions are present in solution. The inversion of the bisignate ECD signal in each case demonstrates the reversible switching of helicity induced by subtle changes in the solvent composition.*

3.3.5. Mass spectrometry studies

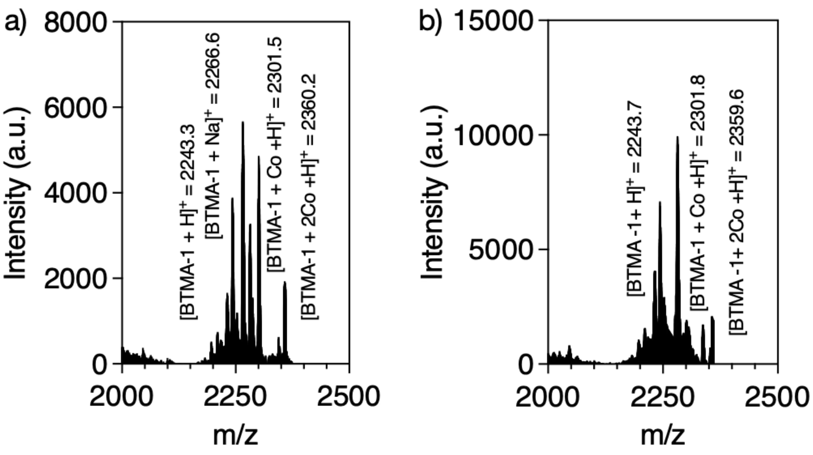

Figure S8. *MALDI spectra of the Co^{II} peptide helicate synthesized in a) HEPES buffer (10 mM, 100 mM NaCl, pH 7.0), ΛΛ-Co$^{II}_2$**BTMA-1**, and in b) DMSO, ΔΔ-Co$^{II}_2$**BTMA-1**.*

MS (MALDI-TOF): Calculated for ΛΛ/ΔΔ-Co$^{II}_2$BTMA-1 $C_{111}H_{123}N_{39}O_{15}Co_2$ = 2359.87. Found (in HEPES buffer): $[BTMA\text{-}1+2Co+H]^+$ = 2360.2; $[BTMA\text{-}1+Co+H]^+$ = 2301.5; $[BTMA\text{-}1+Na]^+$ = 2266.6; $[BTMA\text{-}1+H]^+$ = 2243.2. Found (in DMSO): $[BTMA\text{-}1+2Co+H]^+$ = 2359.6; $[BTMA\text{-}1+Co+H]^+$ = 2301.8; $[BTMA\text{-}1+H]^+$ = 2243.7.

3.3.6. *ECD spectra of **BTMA-1**, **BTMA-1-D** and their Co^{II} peptide helicates in HEPES buffer*

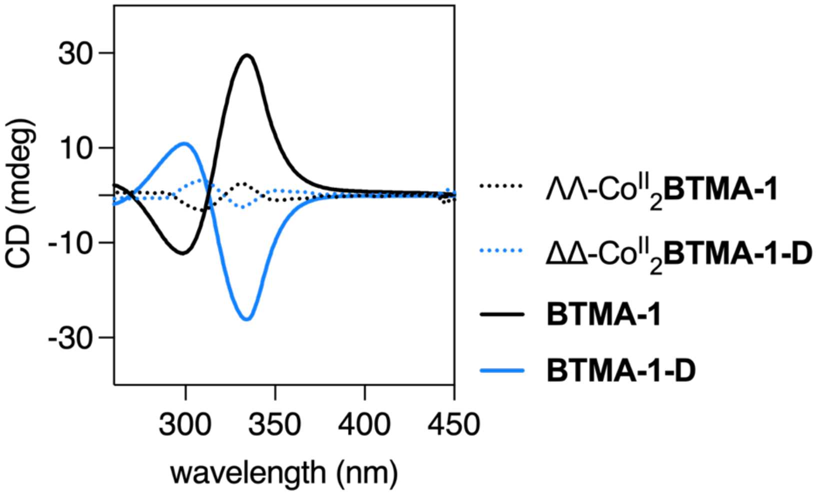

Figure S9. *ECD spectra of **BTMA-1** (black solid line), **BTMA-1-D** (blue solid line), and their corresponding Co^{II} peptide helicates, ΛΛ-Co^{II}_2**BTMA-1** (black dotted line) and ΔΔ-Co^{II}_2**BTMA-1-D** (blue dotted line), recorded in HEPES buffer (10 mM, 100 mM NaCl, pH 7.0). All samples contain 50 µM of the peptide ligand; in the case of the helicates, 15 equivalents of Co^{II} ions were added. The spectra exhibit nearly perfect mirror-image Cotton effects, confirming the enantiomeric relationship between the two peptide ligands and the effective transfer of point chirality from the arginine residues to the overall helicate structure.*

3.4. Synthesis of the discrete ΛΛ-Co^{III} peptide helicate derived from BTMA-1

3.4.1. *In HEPES buffer*

A dispersion of *P*-agg ([**BTMA-1**] = 200 µM) in HEPES buffer (10 mM, 100 mM NaCl, pH 7.0) was incubated with 15 equivalents of $Co(ClO_4)_2{\cdot}6H_2O$ (3 mM) for 20 minutes. Thereafter, the sample was treated with 1.1 eq. of $(NH_4)_2Ce(NO_3)_6$ (3.3 mM) with respect to Co^{II}. After 30 min of reaction, the obtained kinetically inert Co^{III} metallopeptide was purified by semipreparative HPLC in reverse phase (0-40% of AcN + 0.1% TFA in 30 min) and freeze dried. The resulting ΛΛ-Co^{III}_2**BTMA-1** helicate was then characterised by UHPLC-MS and MALDI-TOF mass spectrometry.

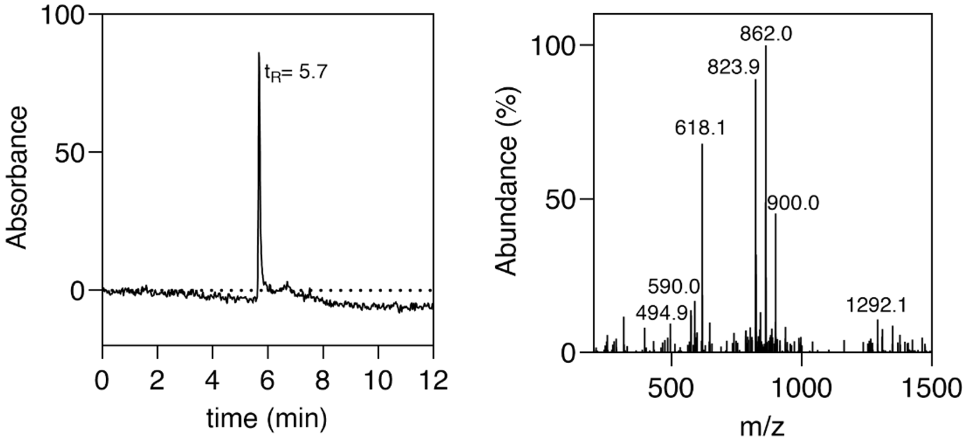

Figure S10. *Left: chromatogram of the purified peptide helicate ΛΛ-Co^{III}_2**BTMA-1**. Right: ESI mass spectra corresponding to the peak with t_R of 5.7 min.*

HPLC-MS (ESI): (5-95% B, t_R= 5.7 min) Calculated for ΛΛ-Co^{III}_2BTMA-1 $C_{111}H_{123}N_{39}O_{15}Co_2$ = 2359.87; found $[M+2H+2TFAH]^{2+}$ = 1292.1; $[M+3H+3TFAH]^{3+}$ = 900.0; $[M+3H+2TFAH]^{2+}$ = 862.0; $[M+3H+TFAH]^{3+}$ = 823.9; $[M+4H+TFAH]^{4+}$ = 618.1; $[M+4H]^{4+}$ = 590.0; $[M+5H+TFAH]^{5+}$ = 494.9.

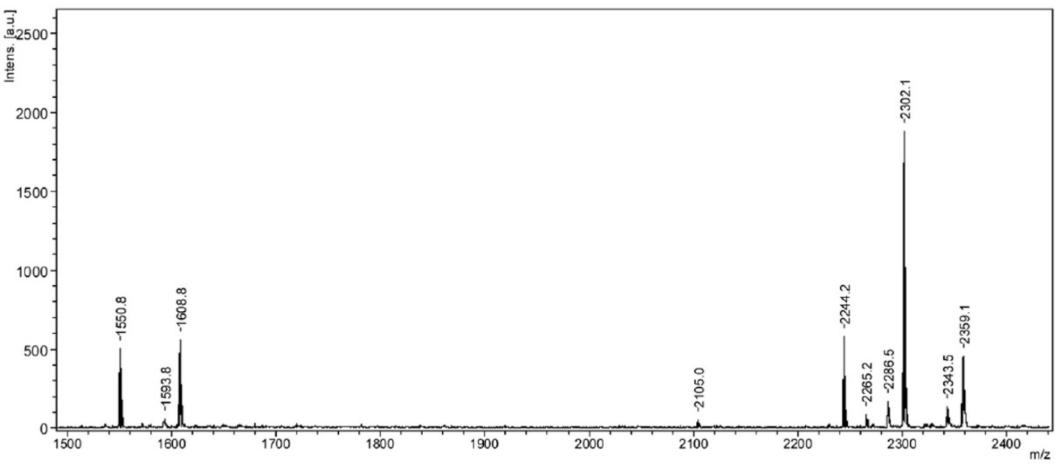

Figure S11. *MALDI spectra of ΛΛ-Co$^{III}_2$**BTMA-1.***

MS (MALDI-TOF): Calculated for ΛΛ-Co$^{III}_2$BTMA-1 $C_{111}H_{123}N_{39}O_{15}Co_2$ = 2359.87; found: $[BTMA\text{-}1+2Co+H]^+$ = 2359.1; $[BTMA\text{-}1+Co+H]^+$ = 2302.1; $[BTMA\text{-}1+H]^+$ = 2244.2

3.4.2. In DMSO

First, it was prepared ΛΛ-Co$^{II}_2$**BTMA-1** by adding 15 equivalents of $Co(ClO_4)_2{\cdot}6H_2O$ to a 250 μM solution of **BTMA-1** in DMSO. The sample was incubated for 1 hour. After that, 1.1 equivalents of $(NH_4)_2Ce(NO_3)_6$ respect to Co^{II} were added and the mixture was stirred for 4 hours. The resulting kinetically inert ΛΛ-Co$^{III}_2$**BTMA-1** derivative was purified by semipreparative HPLC in reverse phase (0-40% of AcN + 0.1% TFA in 30 min) and freeze dried. It was then characterised by UHPLC-MS mass spectrometry.

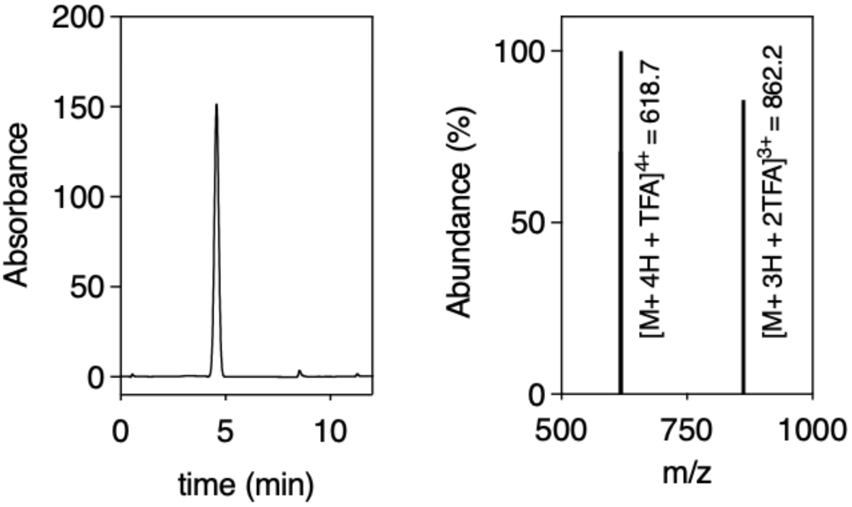

Figure S12. *Left: chromatogram of the purified peptide helicate ΛΛ-Co$^{III}_2$**BTMA-1**. Right: mass spectra corresponding to the peak with t_R of 4.8 min.*

HPLC-MS (ESI): (5-95% B, t_R= 4.8 min) Calculated for ΛΛ-Co$^{III}_2$**BTMA-1** $C_{111}H_{123}N_{39}O_{15}Co_2$ = 2359.87; found $[M+3H+2TFAH]^{2+}$ = 862.2; $[M+4H+TFAH]^{4+}$ = 618.7.

3.5. Fluorescence titration experiments with fluorescein-labelled DNA

*3.5.1. dsDNA-FAM with ΛΛ-Co$^{II}_2$**BTMA-1***

A fluorescence titration was performed (in triplicate) in which, over a 2 μM solution of fluorescein-labelled dsDNA (dsDNA-FAM) aliquots of a stock solution of ΛΛ-Co$^{II}_2$**BTMA-1** in HEPES buffer were added. The K_D of the interaction could not be calculated as a precipitation process occurs in the titration cuvette during the experiment. This result demonstrates the absence of specific interaction between the peptide helicate and dsDNA.

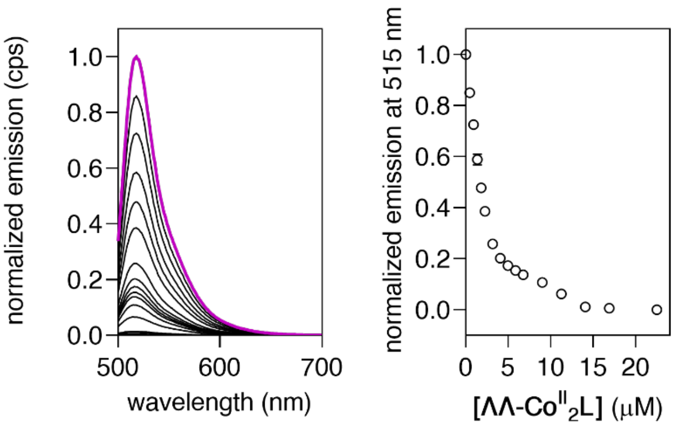

Figure S13. *Left: normalised emission spectra of the 2 μM solution of dsDNA-FAM in absence (purple line) and in the presence of increasing concentrations of ΛΛ-Co$^{II}_{2}$**BTMA-1** (black lines of decreasing intensity). Right: profile at 515 nm of the same fluorescence titration experiment. Conditions: 10 mM HEPES buffer, 100 mM NaCl, pH 7.0; λ_{exc} = 490 nm. dsDNA-FAM oligonucleotide sequences used in this experiment are described in Section 2.4 of this file.*

*3.5.2. dsDNA-FAM with ΛΛ-Co$^{III}_{2}$**BTMA-1***

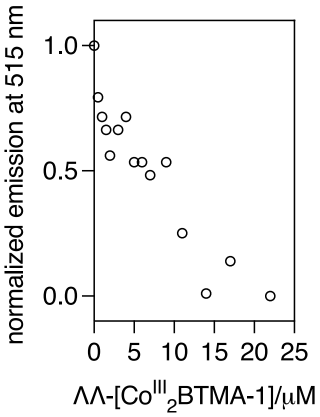

Figure S14. *Normalized emission profile at 515 nm of the titration of a 2 μM solution of dsDNA-FAM with increasing concentrations of ΛΛ-Co$^{III}_{2}$**BTMA-1**. Conditions: 10 mM HEPES buffer, 100 mM NaCl, pH 7.0; λ_{exc} = 490 nm. dsDNA-FAM oligonucleotide sequences used in this experiment are described in Section 2.4 of this file.*

*3.5.3. 3WJ-FAM with ΛΛ-Co$^{III}_{2}$**BTMA-1***

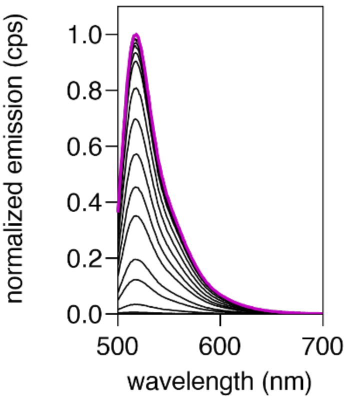

Figure S15. *Full emission spectra of a 2 μM solution of the fluorescein-labelled 3WJ (3WJ-FAM) in absence (purple line) and in the presence of increasing concentrations of ΛΛ-Co$^{III}_{2}$**BTMA-1** (black lines of decreasing intensity). Conditions: 10 mM HEPES buffer, 100 mM NaCl, pH 7.0; λ_{exc} = 490 nm. 3WJ-FAM oligonucleotide sequences used in this experiment are described in Section 2.4 of this file.*

3.5.4. Kinetic 3WJ recognition experiment of P-agg

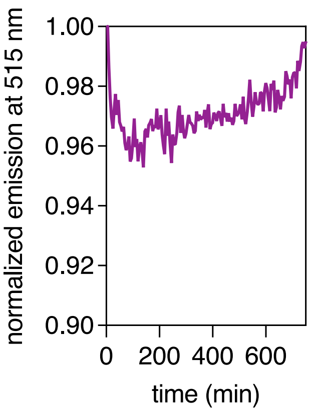

Figure S16. *Normalized fluorescence emission intensity at 515 nm of a 2 µM solution of 3WJ labelled with FAM (3WJ-FAM) in HEPES buffer (10 mM, 100 mM NaCl, pH 7.0) after the addition of 3.75 eq of P-agg at time 0 and then every 5 minutes overnight. λ_{exc}= 490 nm. 3WJ-FAM oligonucleotide sequences used in this experiment are described in Section 2.4 of this file.*

3.6. Gel electrophoresis: DNA binding studies with ΛΛ-Co$^{II}_2$BTMA-1 and ΛΛ-Co$^{III}_2$BTMA-1

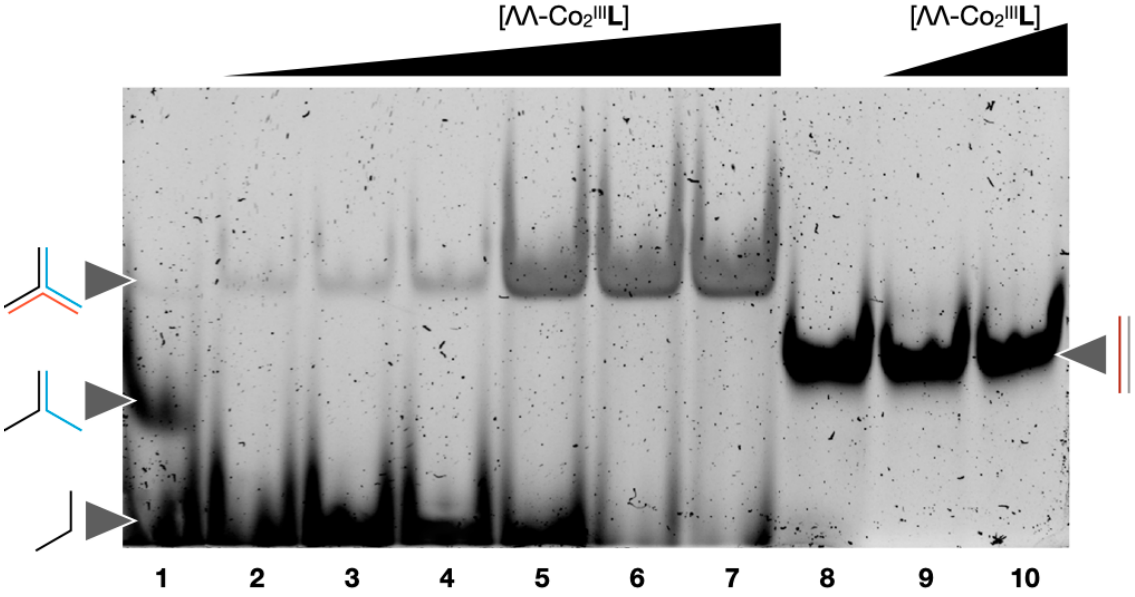

Figure S17. *EMSA DNA binding studies for ΛΛ-Co$^{III}_2$**BTMA-1**. Lanes 1-7: 200 nM of 3WJ with 0, 25, 50, 100, 250, 500 and 750 nM of the peptide helicate, respectively. Lanes 8-10: 50 nM of dsDNA with 0, 500 and 1000 nM of the peptide helicate, respectively. 3WJ and dsDNA oligonucleotide sequences used in these experiments are indicated in Section 2.4 of this file.*

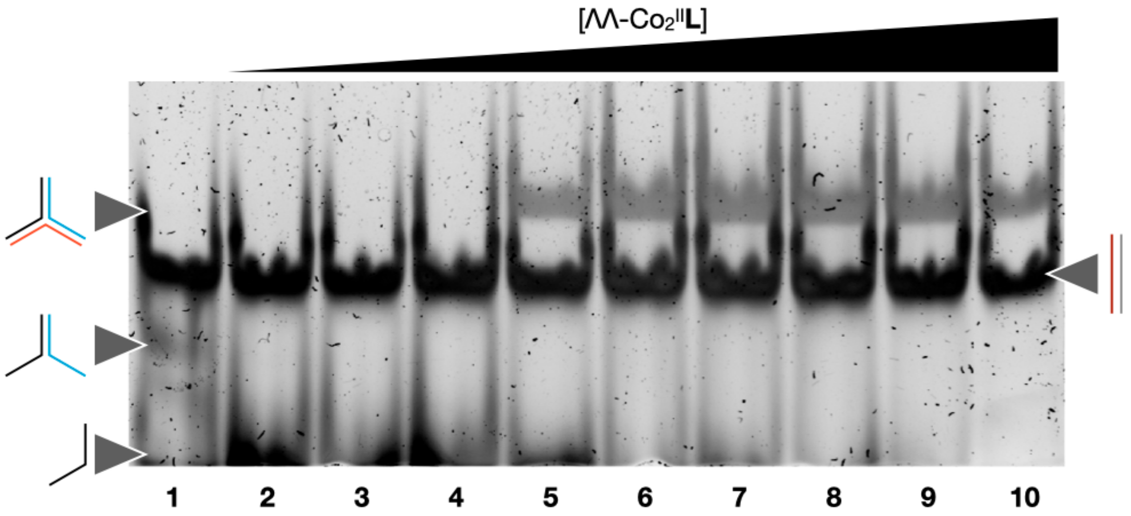

Figure S18. *EMSA competition DNA binding studies for ΛΛ-Co^{II}_2**BTMA-1**. Lanes 1-10: 200 nM of 3WJ plus 200 nM of dsDNA with 0, 25, 50, 100, 250, 500, 750, 1000, 1500 y 2000 nM of the peptide helicate, respectively. 3WJ and dsDNA oligonucleotide sequences used in these experiments are indicated in Section 2.4 of this file.*

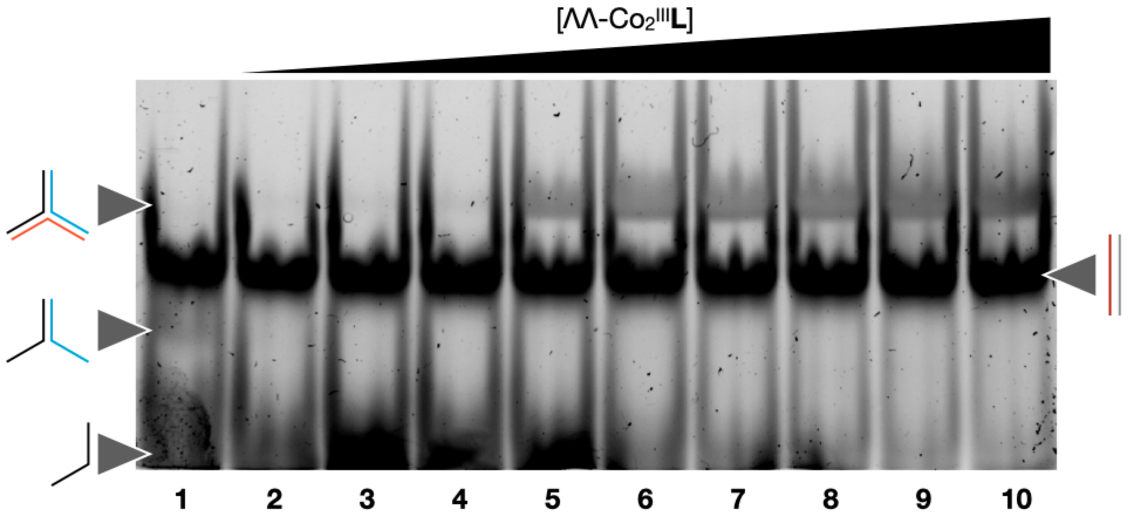

Figure S19. *EMSA competition DNA binding studies for ΛΛ-Co^{III}_2**BTMA-1**. Lanes 1-10: 200 nM of 3WJ plus 200 nM of dsDNA with 0, 25, 50, 100, 250, 500, 750, 1000, 1500 y 2000 nM of the peptide helicate, respectively. 3WJ and dsDNA oligonucleotide sequences used in these experiments are indicated in Section 2.4 of this file.*

3.7. TEM studies

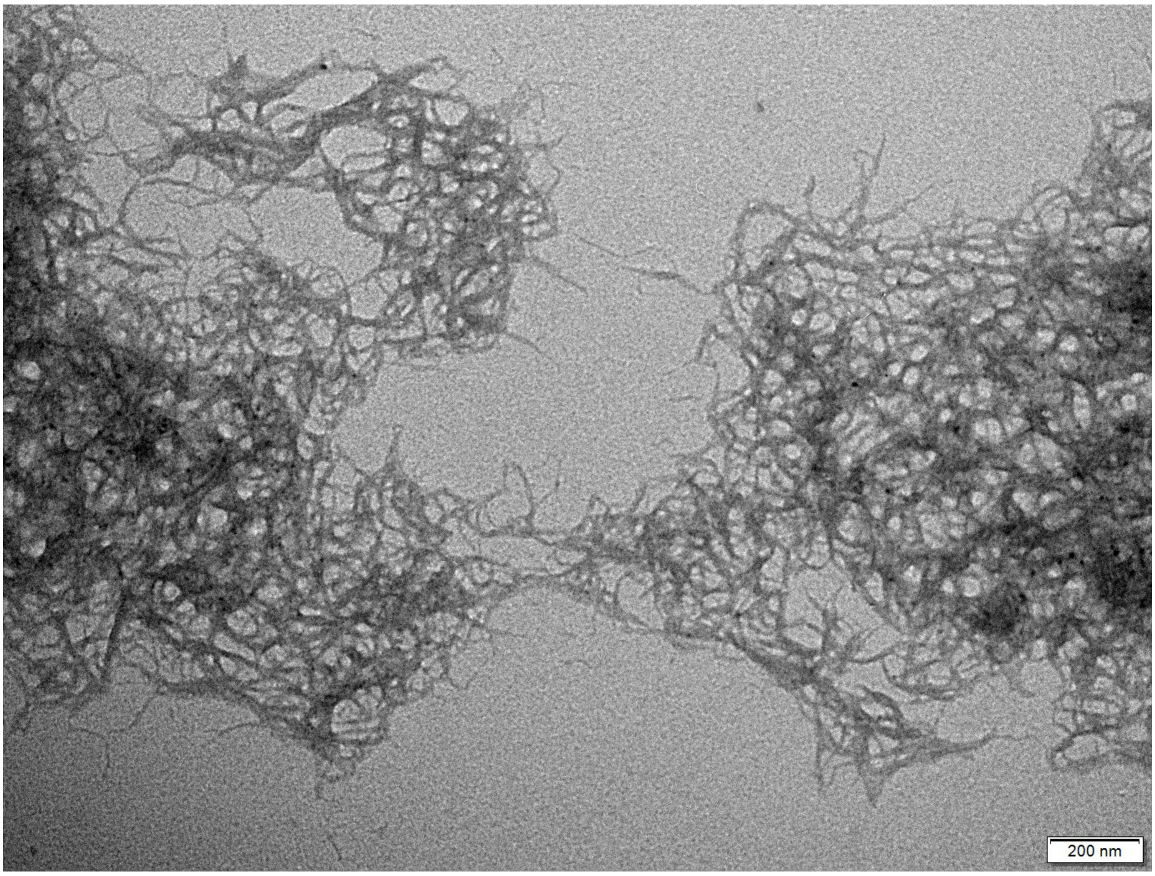

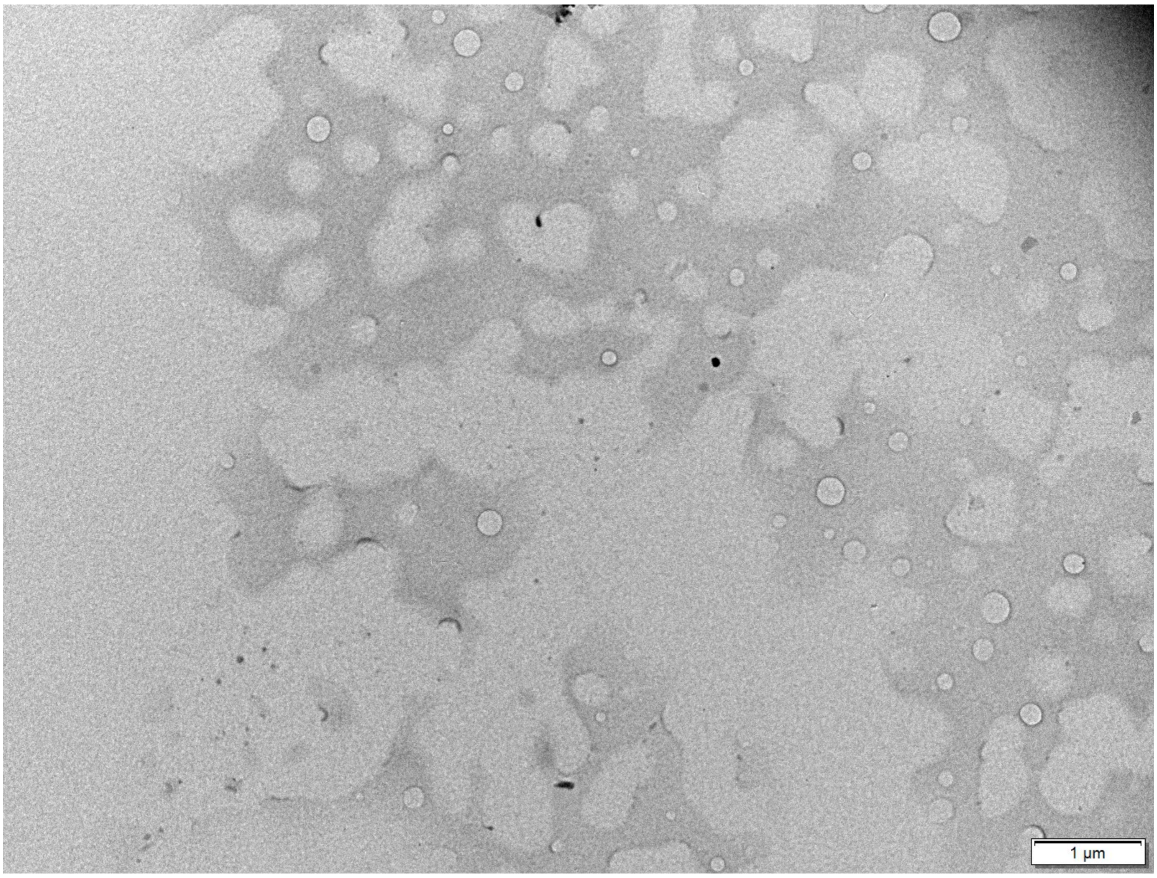

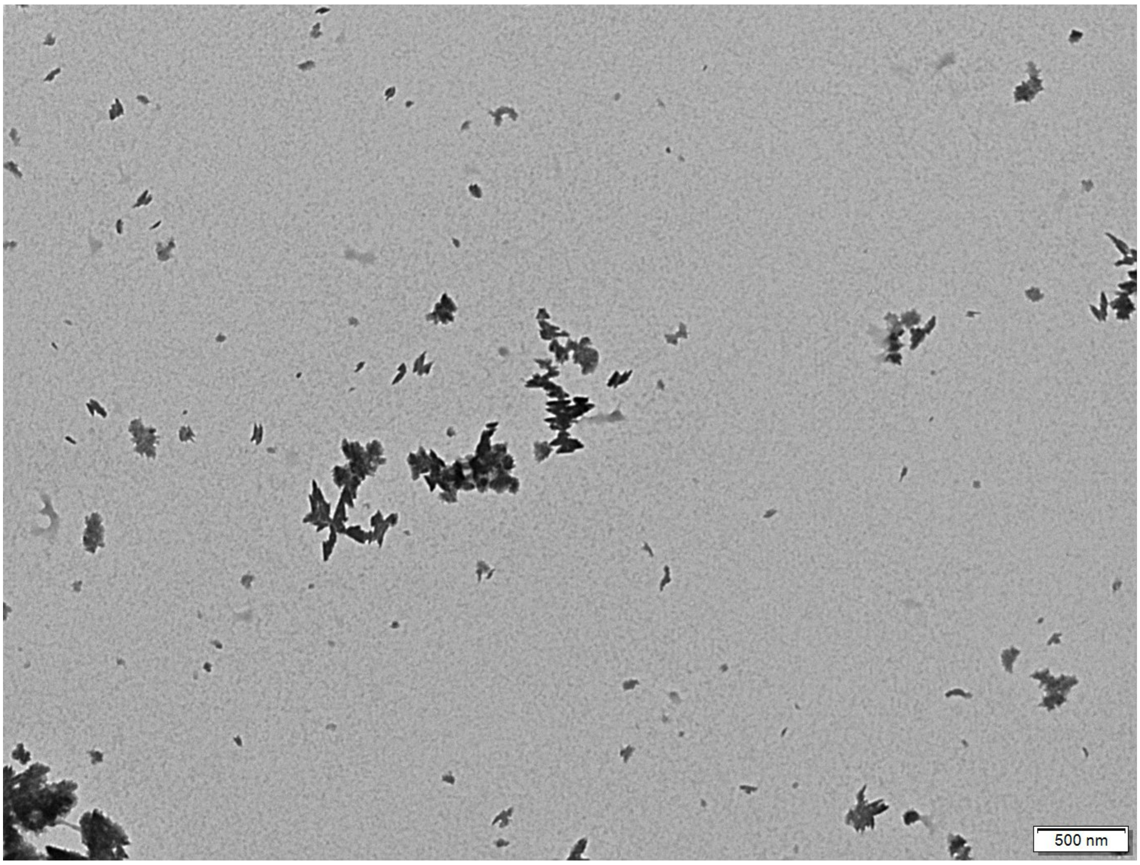

Figure S20. *Top, TEM image of a 10 µM solution of **BTMA-1** in HEPES buffer (10 mM, 100 mM NaCl, pH= 7.0), showing the formation of very elongated fibres with an average width of 18 nm. Middle, TEM image of a 10 µM solution of **BTMA-1** in HEPES buffer in the presence of 15 equivalents of Co^{II} ions, where no fibres are observed. Bottom, TEM image of a 10 µM solution of **BTMA-1** in DMSO, where no fibres are observed.*

3.8. Melting ECD studies on *P*-agg

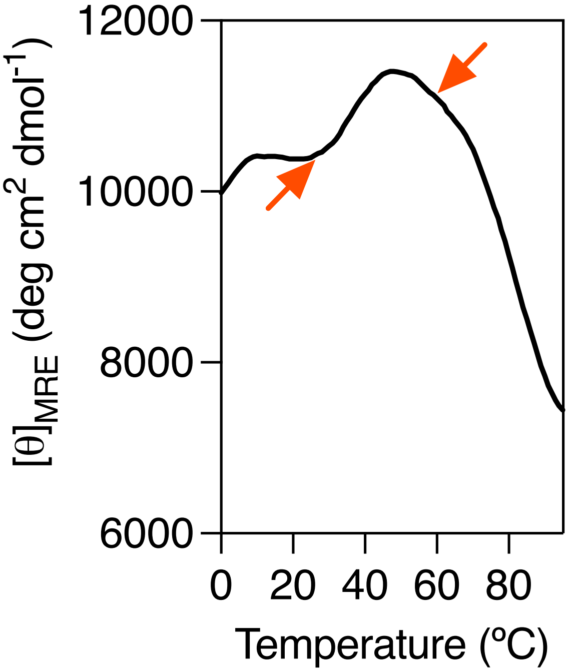

Figure S21. *ECD melting profile at 320 nm measured by increasing the temperature of a dispersion of P-agg ([**BTMA-1**] = 10 µM) in HEPES buffer (10 mM, 100 mM NaCl, pH 7.0) from 0 to 95°C. The melting profile shows two transitions (orange arrows) around 25 and 60°C which can be ascribed to the aggregation and disaggregation processes, respectively.*

3.9. Solvent denaturation studies of *P*-agg

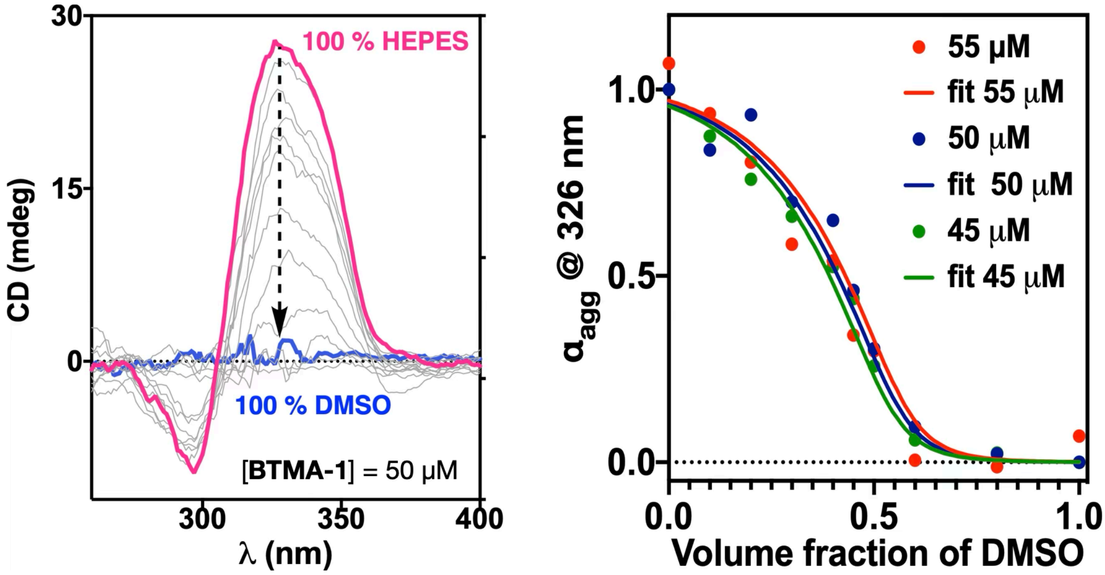

Figure S22. *Left, ECD solvent denaturation studies of P-agg ([**BTMA-1**] = 50 µM). Right, denaturation curves, with the corresponding fittings, of P-agg ([**BTMA-1**] = 45, 50 and 55 µM).*

3.10. Molecular modeling studies

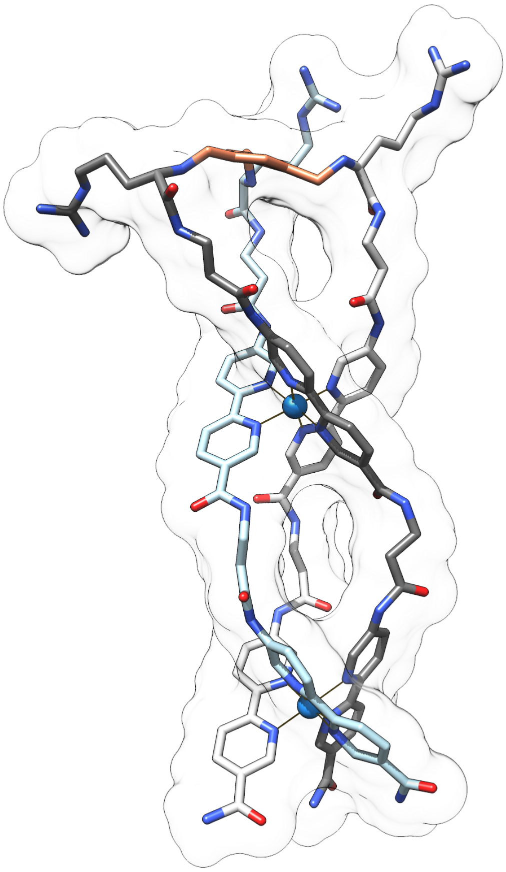

Figure S23. *Energy-minimized structure of chiral peptide helicate ΛΛ-Co$^{II}_{2}$**BTMA-1**, calculated using water as solvent. The experimental data for these studies can be found in Section 2.10 of this file.*

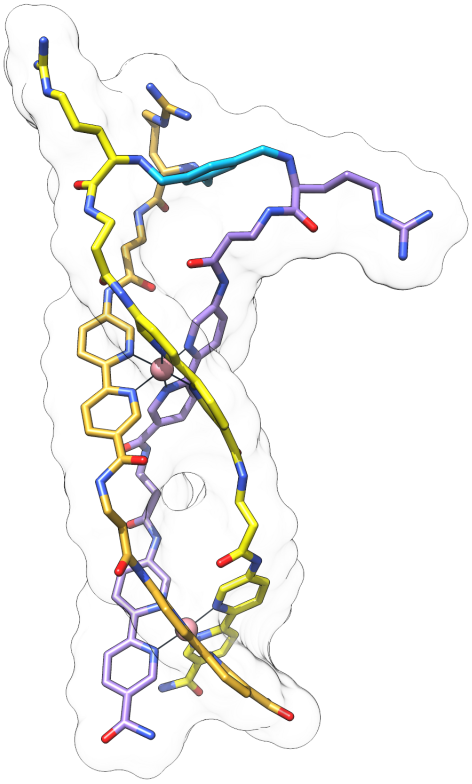

Figure S24. *Energy-minimized structure of chiral peptide helicate ΛΛ-Co$^{III}_{2}$**BTMA-1**, calculated using water as solvent. The experimental data for these studies can be found in Section 2.10 of this file.*

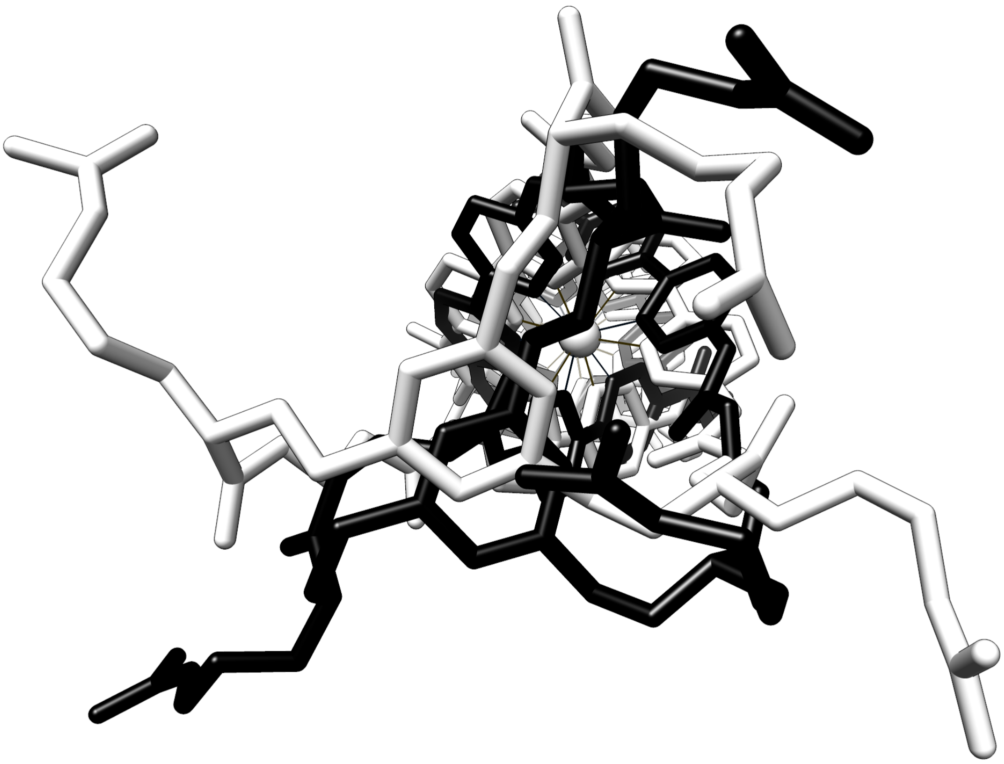

Figure S25. *Comparative view of the energy-minimized structures of ΛΛ-Co$^{II}_2$**BTMA-1** (white atoms) and ΛΛ-Co$^{III}_2$**BTMA-1** (black atoms), calculated using water as solvent. The experimental data for these studies can be found in Section 2.10 of this file.*

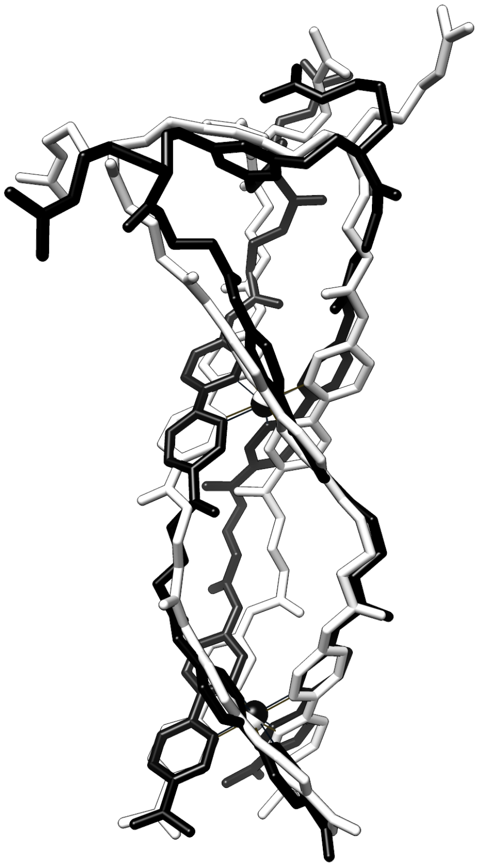

Figure S26. *Alternative comparative view of the energy-minimized structures of ΛΛ-Co$^{II}_2$**BTMA-1** (white atoms) and ΛΛ-Co$^{III}_2$**BTMA-1** (black atoms), calculated using water as the solvent. The experimental data for these studies can be found in Section 2.10 of this file.*

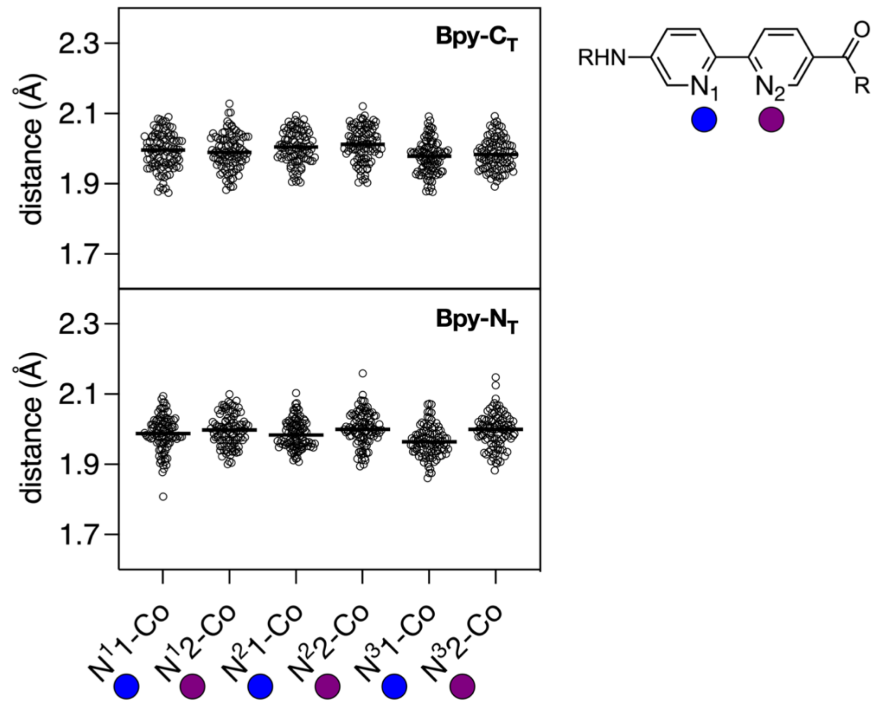

Figure S27. *Summary of the Co-N bond distances at the C- and N-terminal Co^{II} coordination sites (Bpy-C$_T$ and Bpy-N$_T$, respectively) found in the different poses of the energy-minimized structure of ΛΛ-Co$^{II}_2$**BTMA-1,** calculated using water as the solvent. Superscripts 1, 2 and 3 refer to the three distinct peptide strands of the helicate. Subscripts 1 and 2 refer to the nitrogen atoms at the N- and C-terminal positions of the Bpy amino acid, respectively.*

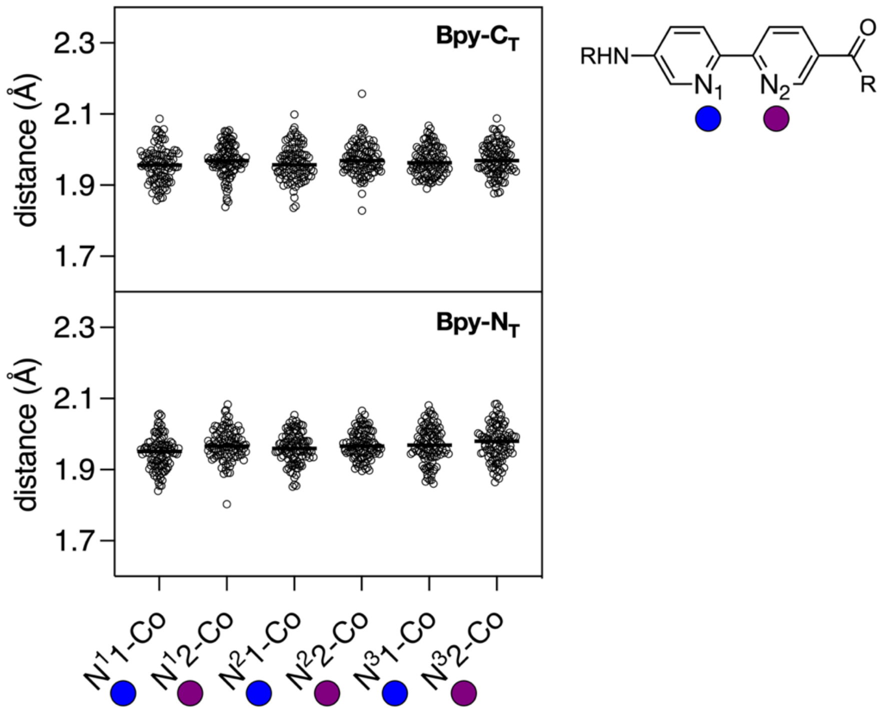

Figure S28. *Summary of the Co-N bond distances at the C- and N-terminal Co^{III} coordination sites (Bpy-C$_T$ and Bpy-N$_T$, respectively) found in the different poses of the energy-minimized structure of ΛΛ-Co$^{III}_2$**BTMA-1,** calculated using water as the solvent. Superscripts 1, 2 and 3 refer to the three distinct peptide strands of the helicate. Subscripts 1 and 2 refer to the nitrogen atoms at the N- and C-terminal positions of the Bpy amino acid, respectively. The slight differences observed relative to the Co^{II} analogue (Figure S27) are consistent with the higher charge-to-radius (Q/r) ratio of Co^{III} compared to Co^{II}.*

	Bpy N_T		Bpy C_T	
	distance (Å)	SD	distance (Å)	SD
**N_1^1-Co**	1.988	0.050	1.992	0.051
**N_2^1-Co**	1.996	0.045	1.992	0.050
**N_1^2-Co**	1.986	0.041	2.004	0.045
**N_2^2-Co**	2.001	0.047	2.012	0.048
**N_1^3-Co**	1.965	0.043	1.976	0.046
**N_2^3-Co**	1.996	0.047	1.986	0.044

Table S3. *Co-N bond distances at the C- and N-terminal Co^{II} coordination sites (Bpy-C_T and Bpy-N_T, respectively) of the energy-minimized structure of ΛΛ-Co^{II}_2**BTMA-1,** calculated using water as the solvent. Superscripts 1, 2 and 3 refer to the three distinct peptide strands of the helicate. Subscripts 1 and 2 refer to the nitrogen atoms at the N- and C-terminal positions of the Bpy amino acid, respectively.*

	Bpy N_T		Bpy C_T	
	distance (Å)	SD	distance (Å)	SD
**N_1^1-Co**	1.948	0.046	1.957	0.049
**N_2^1-Co**	1.971	0.044	1.970	0.045
**N_1^2-Co**	1.961	0.044	1.962	0.048
**N_2^2-Co**	1.971	0.039	1.973	0.044
**N_1^3-Co**	1.970	0.050	1.967	0.040
**N_2^3-Co**	1.976	0.049	1.972	0.045

Table S4. *Co-N bond distances at the C- and N-terminal Co^{III} coordination sites (Bpy-C_T and Bpy-N_T, respectively) of the energy-minimized structure of ΛΛ-Co^{III}_2**BTMA-1,** calculated using water as the solvent. Superscripts 1, 2 and 3 refer to the three distinct peptide strands of the helicate. Subscripts 1 and 2 refer to the nitrogen atoms at the N- and C-terminal positions of the Bpy amino acid, respectively.*

	Bpy N_T		Bpy C_T	
	angle (Å)	SD	angle (Å)	SD
**N_1^1-Co-N_1^2**	97.49	1.68	97.64	1.74
**N_1^1-Co-N_3^2**	87.40	1.93	87.07	1.99
**N_1^1-Co-N_1^3**	96.16	1.71	95.57	1.71
**N_1^2-Co-N_1^3**	97.03	1.78	96.42	1.58
**N_1^2-Co-N_3^3**	86.87	2.08	86.91	2.03
**N_1^3-Co-N_3^1**	88.26	2.02	87.34	2.01
**N_3^1-Co-N_3^2**	96.95	1.77	98.38	1.90
**N_3^2-Co-N_3^3**	97.59	1.95	97.32	1.76
**N_3^3-Co-N_3^1**	97.66	1.90	97.45	1.82

Table S5. *N-Co-N bond angles at the C- and N-terminal Co^{II} coordination sites (Bpy-C_T and Bpy-N_T, respectively) of the energy-minimized structure of ΛΛ-Co^{II}_2**BTMA-1,** calculated using water as the solvent. Superscripts 1, 2 and 3 refer to the three distinct peptide strands of the helicate. Subscripts 1 and 2 refer to the nitrogen atoms at the N- and C-terminal positions of the Bpy amino acid, respectively.*

	Bpy N_T		Bpy C_T	
	angle (Å)	SD	angle (Å)	SD
**N_1^1-Co-N_1^2**	98.07	1.48	97.82	1.71
**N_1^1-Co-N_3^2**	86.36	2.00	86.16	1.99
**N_1^1-Co-N_1^3**	97.90	1.80	97.88	1.70
**N_1^2-Co-N_1^3**	97.96	1.72	98.09	1.70
**N_1^2-Co-N_3^3**	86.15	1.89	86.20	1.84
**N_1^3-Co-N_3^1**	86.48	2.31	86.14	1.98
**N_3^1-Co-N_3^2**	97.39	1.89	97.85	1.83
**N_3^2-Co-N_3^3**	97.43	1.73	97.74	1.87
**N_3^3-Co-N_3^1**	97.58	1.93	97.44	1.50

Table S6. *N-Co-N bond angles at the C- and N-terminal Co^{III} coordination sites (Bpy-C_T and Bpy-N_T, respectively) of the energy-minimized structure of ΛΛ-Co^{III}_2**BTMA-1,** calculated using water as the solvent. Superscripts 1, 2 and 3 refer to the three distinct peptide strands of the helicate. Subscripts 1 and 2 refer to the nitrogen atoms at the N- and C-terminal positions of the Bpy amino acid, respectively.*

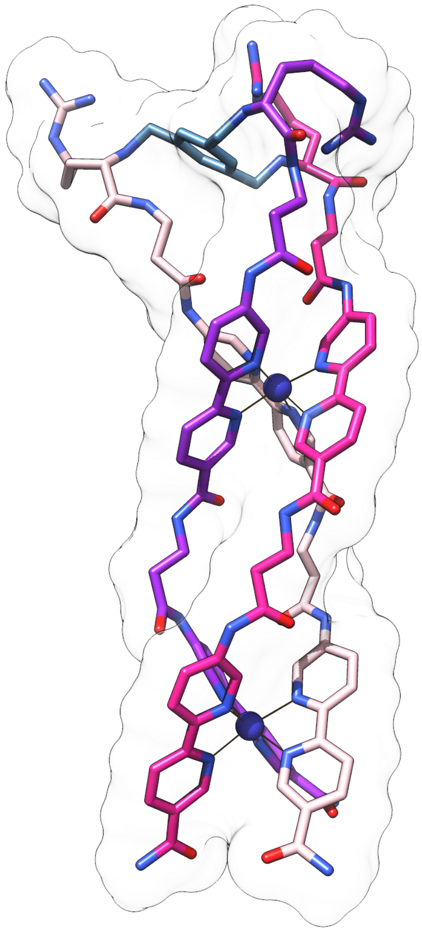

Figure S29. *Energy-minimized structure of chiral peptide helicate ΔΔ-Co$^{II}_{2}$**BTMA-1**, calculated using DMSO as solvent. The experimental data for these studies can be found in Section 2.10 of this file.*

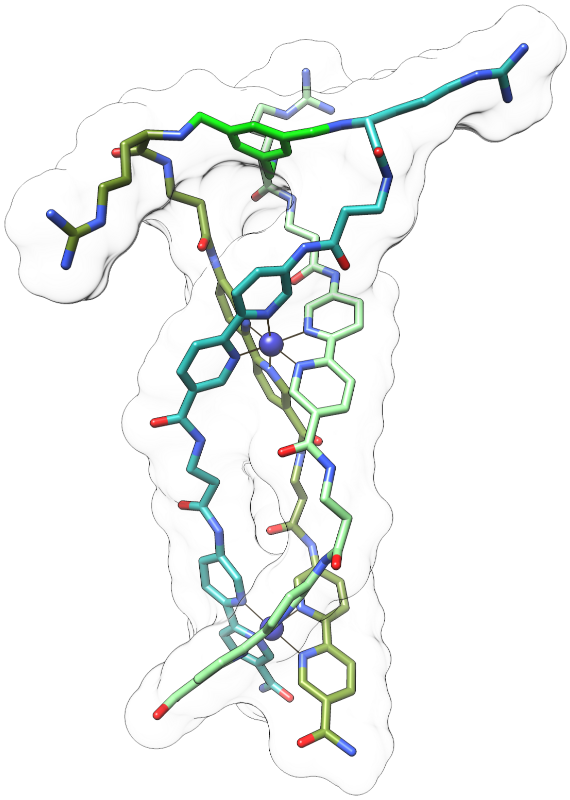

Figure S30. *Energy-minimized structure of chiral peptide helicate ΔΔ-Co$^{II}_{2}$**BTMA-1-D**, calculated using water as solvent. The experimental data for these studies can be found in Section 2.10 of this file.*

	Bpy N_T		Bpy C_T	
	distance (Å)	SD	distance (Å)	SD
**N_1^1-Co**	2.025	0.047	2.038	0.054
**N_2^1-Co**	2.178	0.080	2.150	0.077
**N_1^2-Co**	1.988	0.043	1.992	0.047
**N_2^2-Co**	2.003	0.043	2.006	0.040
**N_1^3-Co**	2.131	0.076	2.143	0.079
**N_2^3-Co**	2.037	0.053	2.037	0.053

Table S7. *Co-N bond distances at the C- and N-terminal Co^{II} coordination sites (Bpy-C_T and Bpy-N_T, respectively) of the energy-minimized structure of ΔΔ-Co^{II}_2**BTMA-1,** calculated using DMSO as the solvent. Superscripts 1, 2 and 3 refer to the three distinct peptide strands of the helicate. Subscripts 1 and 2 refer to the nitrogen atoms at the N- and C-terminal positions of the Bpy amino acid, respectively.*

	Bpy N_T		Bpy C_T	
	angle (Å)	SD	angle (Å)	SD
**N_1^1-Co-N_1^2**	98.17	1.91	97.51	2.35
**N_1^1-Co-N_3^3**	89.49	2.11	89.23	2.11
**N_1^1-Co-N_1^3**	99.32	2.91	99.95	2.92
**N_1^2-Co-N_1^3**	96.97	2.28	97.99	2.54
**N_1^2-Co-N_3^1**	90.22	2.77	89.02	2.70
**N_1^3-Co-N_3^2**	90.85	3.22	87.99	3.32
**N_3^1-Co-N_3^2**	96.38	2.59	98.63	2.75
**N_3^2-Co-N_3^3**	97.11	2.17	97.75	1.74
**N_3^3-Co-N_3^1**	99.77	2.85	99.86	2.52

Table S8. *N-Co-N bond angles at the C- and N-terminal Co^{III} coordination sites (Bpy-C_T and Bpy-N_T, respectively) of the energy-minimized structure of ΔΔ-Co^{III}_2**BTMA-1,** calculated using DMSO as the solvent. Superscripts 1, 2 and 3 refer to the three distinct peptide strands of the helicate. Subscripts 1 and 2 refer to the nitrogen atoms at the N- and C-terminal positions of the Bpy amino acid, respectively.*

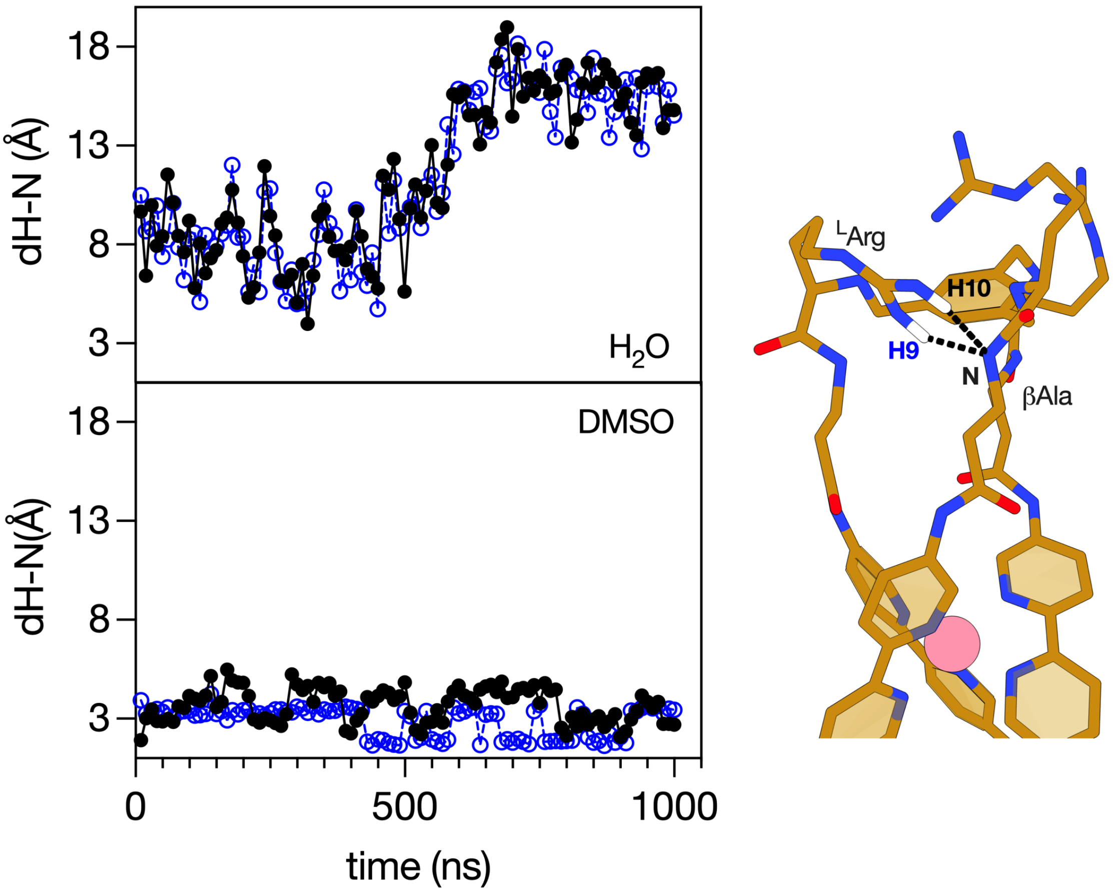

Figure S31. *Comparison of the time evolution of the distances (in Å) between the H9 proton of the L-Arg residue from one strand and the amide nitrogen of the β-Ala residue from an adjacent strand (blue circles), and between the H10 proton of the same L-Arg and the same amide nitrogen atom (black circles), over 1000 ns of molecular dynamics simulations. The data correspond to the energy-minimized structures of the ΛΛ-Co^{II}**BTMA-1** helicate in water (top) and the ΔΔ-Co^{II}**BTMA-1** helicate in DMSO (bottom). A representative intramolecular hydrogen bonding interaction is shown on the right.*

3.11. NMR studies

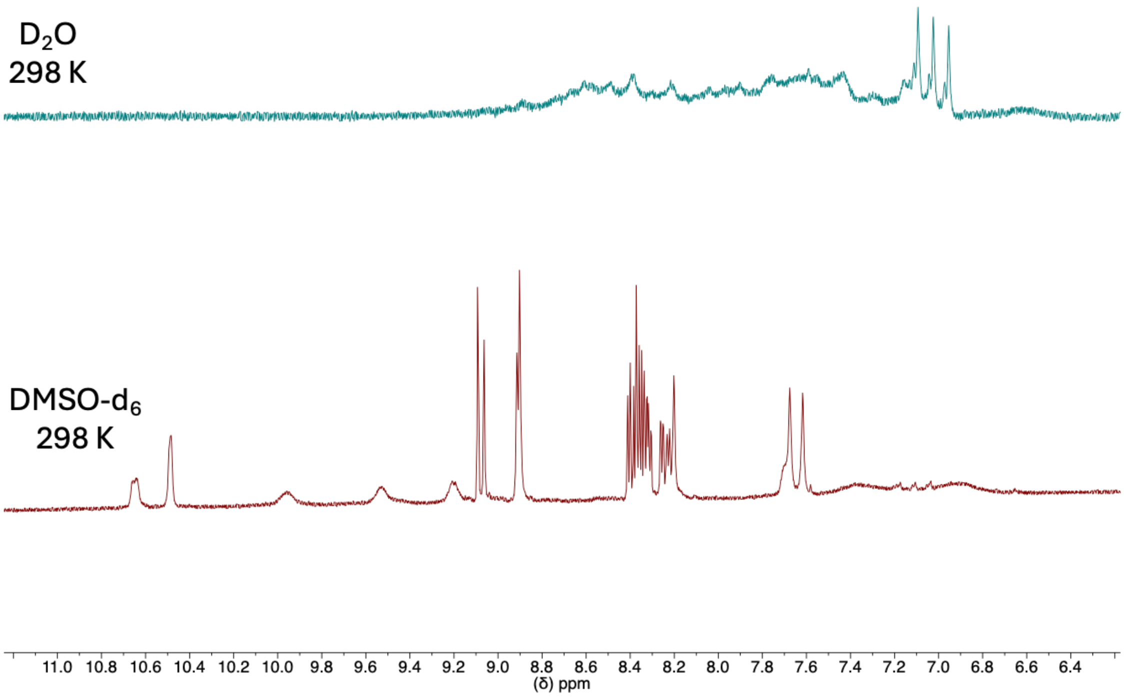

Figure S32. *Comparison of the ^{1}H NMR spectra of* ***BTMA-1*** *in D_2O and DMSO showing aggregated and molecularly dissolved states, respectively (750 MHz, 298 K).*

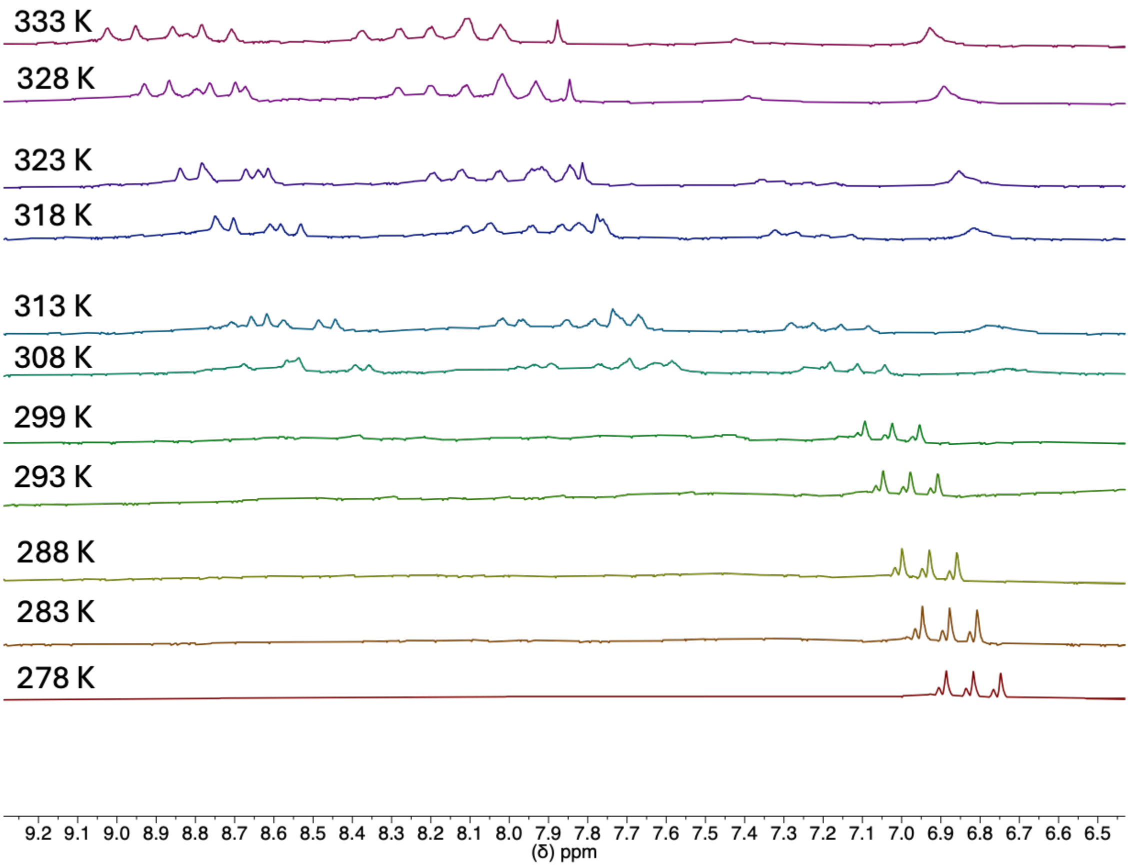

Figure S33. *VT-^{1}H NMR spectra of* ***BTMA-1*** *in D_2O (750MHz).*

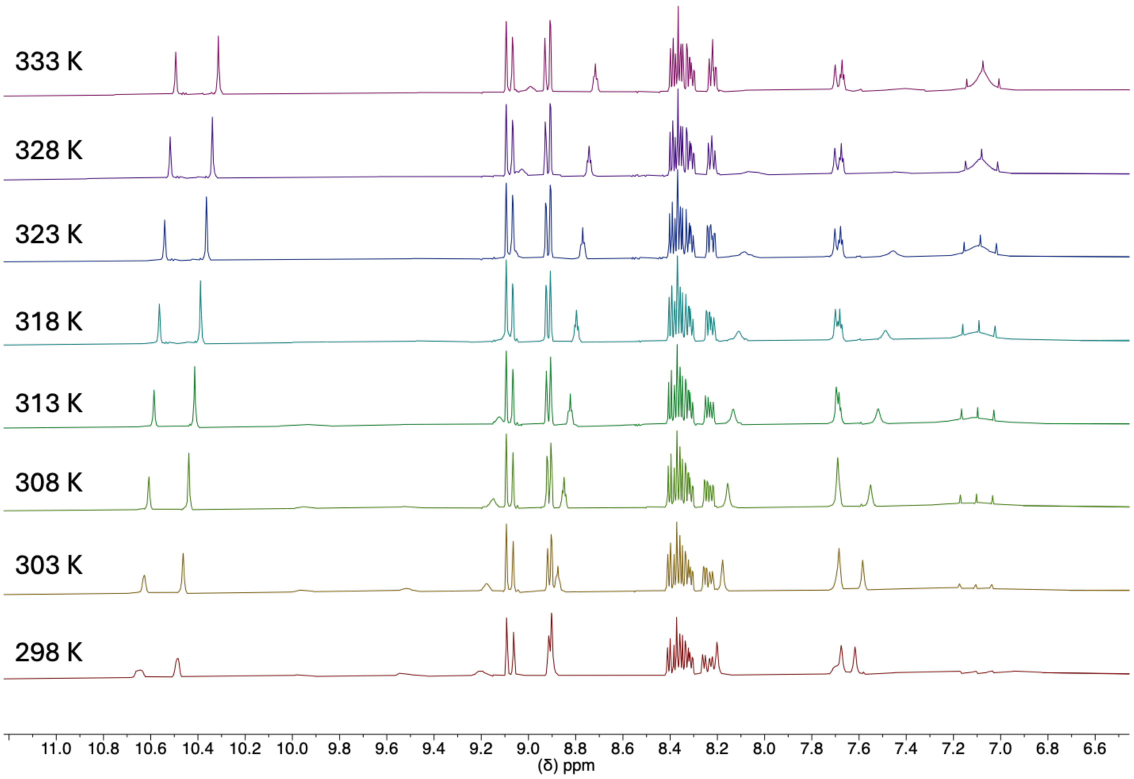

Figure S34. *VT-^{1}H NMR spectra of **BTMA-1** in D_2O (750MHz).*
